# A new xinjiangchelyid turtle from the Middle Jurassic of Xinjiang, China and the evolution of the basipterygoid process in Mesozoic turtles

**DOI:** 10.1186/1471-2148-13-203

**Published:** 2013-09-22

**Authors:** Márton Rabi, Chang-Fu Zhou, Oliver Wings, Sun Ge, Walter G Joyce

**Affiliations:** 1 Institut für Geowissenschaften, University of Tübingen, Hölderlinstraße 12, 72074, Tübingen, Germany; 2 Department of Paleontology & MTA–ELTE Lendület Dinosaur Research Group, Eötvös Loránd University, Budapest, Hungary; 3 Paleontological Institute, Shenyang Normal University, 253 North Huanghe Street, Shenyang 110034, China; 4 Niedersächsisches Landesmuseum Hannover, 5 Willy-Brandt-Allee, Hannover 530169, Germany; 5 Yale Peabody Museum of Natural History, 170 Whitney Avenue, New Haven, Connecticut 06511, USA

## Abstract

**Background:**

Most turtles from the Middle and Late Jurassic of Asia are referred to the newly defined clade Xinjiangchelyidae, a group of mostly shell-based, generalized, small to mid-sized aquatic froms that are widely considered to represent the stem lineage of Cryptodira. Xinjiangchelyids provide us with great insights into the plesiomorphic anatomy of crown-cryptodires, the most diverse group of living turtles, and they are particularly relevant for understanding the origin and early divergence of the primary clades of extant turtles.

**Results:**

Exceptionally complete new xinjiangchelyid material from the ?Qigu Formation of the Turpan Basin (Xinjiang Autonomous Province, China) provides new insights into the anatomy of this group and is assigned to *Xinjiangchelys wusu* n. sp. A phylogenetic analysis places *Xinjiangchelys wusu* n. sp. in a monophyletic polytomy with other xinjiangchelyids, including *Xinjiangchelys junggarensis*, *X*. *radiplicatoides*, *X. levensis* and *X. latiens*. However, the analysis supports the unorthodox, though tentative placement of xinjiangchelyids and sinemydids outside of crown-group Testudines. A particularly interesting new observation is that the skull of this xinjiangchelyid retains such primitive features as a reduced interpterygoid vacuity and basipterygoid processes.

**Conclusions:**

The homology of basipterygoid processes is confidently demonstrated based on a comprehensive review of the basicranial anatomy of Mesozoic turtles and a new nomenclatural system is introduced for the carotid canal system of turtles. The loss of the basipterygoid process and the bony enclosure of the carotid circulation system occurred a number of times independently during turtle evolution suggesting that the reinforcement of the basicranial region was essential for developing a rigid skull, thus paralleling the evolution of other amniote groups with massive skulls.

## Background

Most recent, morphology-based, phylogenetic studies of fossil and extant turtles agree that the Middle to Late Jurassic was a particularly important phase in the early diversification of crown group Testudines [1-6]. Xinjiangchelyidae is a clade of turtles that includes some of the most common taxa known from this time period in Asia and that is widely considered to represent the primitive morphology of the cryptodiran stem lineage [2-4,7-16]. The exact content of this clade is still an open question, however, as the anatomy and phylogenetic relationships of many candidate taxa are still poorly known.

A new species of xinjiangchelyid, *Xinjiangchelys wusu* n. sp., is described here on the basis of exceptionally well preserved skeletons that were found and recovered by the 2009 and 2011 Field Teams of the Sino-German Cooperation Project in the Upper Jurassic ?Qigu Formation of the Turpan Basin, Xinjiang Autonomous Province, China and that provide new insights into the morphology of xinjiangchelyids.

One anatomical region of special interest for turtle evolution is the basicranium. The basisphenoid of some paracryptodires and xinjiangchelyids, including *Xinjiangchelys wusu* n. sp., has previously been shown to exhibit a pair of lateral processes that were homologized with the basipterygoid process of basal amniotes [15,17]. However, the homology of these structures is controversial in the literature [18-20] and a comprehensive assessment of this issue is still outstanding. We here identify similar basisphenoid processes in a broad range of extinct turtles and conclude that their presence has been overlooked in the Mesozoic turtle literature during the last forty years. We here furthermore provide compelling morphological evidence for the homology of the basisphenoid processes of xinjiangchelyids with the basipterygoid processes of basal turtles and basal amniotes and review the evolution of this structure in Mesozoic turtles. We finally present an internally consistent nomenclatural system that reflects recent insights into the morphology of the carotid canal system. To test the phylogenetic implications of our new insights, we analyzed an extensive sample of xinjiangchelyids in a global, cladistic framework of turtles. We obtained the unorthodox placement of this clade outside crown group Testudines, which may hint at a surprisingly extensive evolutionary history of the turtle stem lineage.

## Methods

### Geological settings

**Figure 1:**
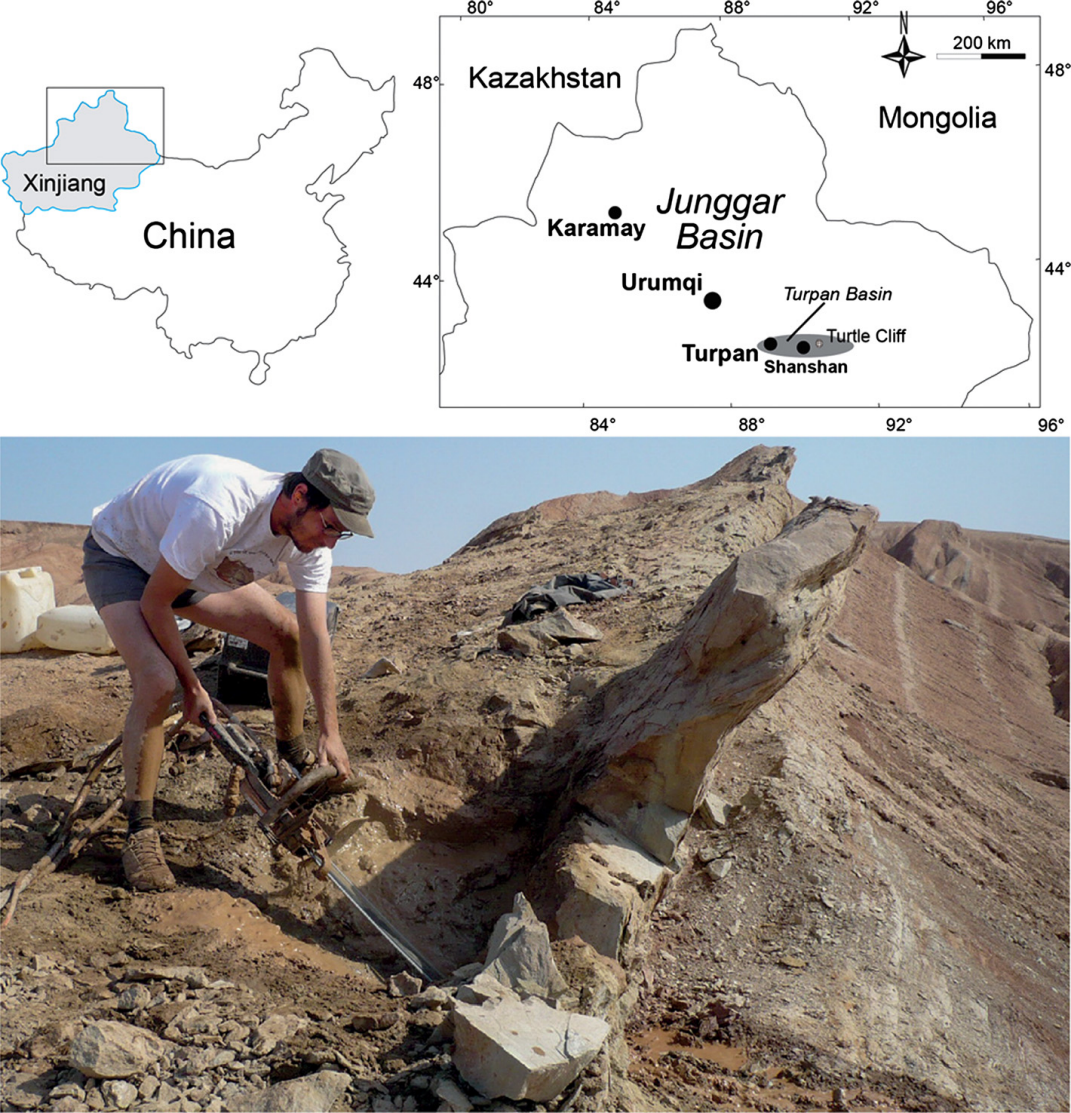
The geographic location of the “Turtle Cliff” site in the Turpan Basin of Xinjiang Autonomous Province, China (above) and a photograph of the cliff where the turtles were found and cut out with the help of a rock saw (below).

The “Turtle Cliff Fossil Site” yielded the new material described herein and is located within the Flaming Mountains about 26 km ENE of the city of Shanshan in the Turpan Basin, Xinjiang Autonomous Province, China (Figure 1). The Flaming Mountains consist of Triassic to Paleogene sediments that were uplifted during the Neogene [21-23]. Published reports on the geology and stratigraphy of the Flaming Mountains in particular and the Turpan Basin in general are rare (e.g., [24] and references therein) and many uncertainties therefore exist regarding the absolute age of formations and their correlation with similar units in other Central Asian basins. Jurassic clastic strata in the Flaming Mountains were preliminarily divided into the Early Jurassic Sangonghe Formation, the Middle Jurassic Xishanyao, Sanjianfang, Qiketai, and Qigu Formations (the latter was recently dated in the Junggar Basin with 164.6 Ma ± 1.4 Ma, [25]), and the Late Jurassic Karaza Formation [26]. Future stratigraphic research needs to clarify whether Late Jurassic strata are indeed mostly absent in the area.

Piedmont-fluvial deposits dominate the upper parts of the Jurassic sequence [27,28]. Red-colored sediments, especially prominent in the Qigu Formation, indicate a reduction in the monsoonal circulation in Asia resulting in a paleoclimatic change from humid to seasonally dry during the late Middle and early Late Jurassic [24,25,28-31]. The total thickness of the supposed Qigu Formation is about 850 m in the area of the Turtle Cliff Fossil Site [31]. The formation is rich in vertebrate fossils, dominated by dinosaurs and turtles [28]. Finds of the latter include the spectacular turtle taphocoenosis at Mesa Chelonia [28] near the lower border of the formation and the herein introduced Turtle Cliff Fossil Site near the base of the upper third of the formation.

The Turtle Cliff Fossil Site is situated geographically 1 km to the ENE and stratigraphically 500 m above the Mesa Chelonia site [28]. Since no explicit justification has been given for the correlation of the strata supposedly belonging to the Qigu Formation in the Turpan Basin, the assignment of rocks units exposed in the Flaming Mountains to this formation is not transparent [28], but our preliminary classification places both sites within the Qigu Formation. The deposits that allegedly represent the Qigu Formation in the Turpan Basin are characterized by alternating coarse and fine-grained sediments that often contain unionid freshwater bivalves, reflecting changing depositional conditions typical of river systems [24,31]. Temporary subaerial exposure is indicated by paleosols [28].

The turtle skeletons at the Turtle Cliff Fossil Site were found on the top of a low hill in a steeply inclined (65°), fine-grained and strongly cemented sandstone layer rich in lithoclasts. Above and below the turtle-bearing sandstone horizon follows a succession of predominately red silt-and mudstones.

### Material studied in this paper

**Figure 2:**
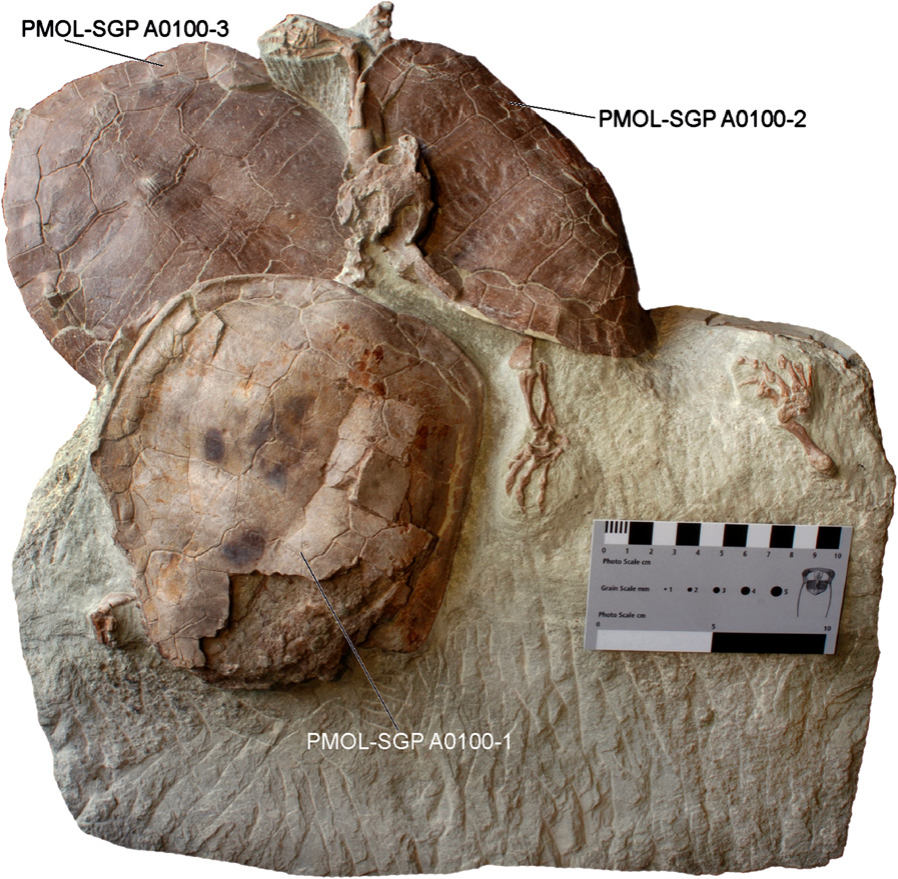
**Type series of *Xinjiangchelys wusu *n. sp. from “Turtle Cliff”, Turpan Basin of Xinjiang Autonomous Province, China, ?Qigu Formation, Middle Jurassic.**

Our description of *Xinjiangchelys wusu* n. sp. is based on a sandstone slab with at least 3 individuals (Figure 2) that were excavated during the 2011 joint field season of the University of Tübingen, Shenyang Normal University, and Jilin University, that was lead and carried out by all coauthors at the Turtle Cliff Fossil Site (see Geological Settings). The quarried fossils are currently housed at the Paleontology Museum of Liaoning (PMOL) at Shenyang Normal University, Shenyang, Liaoning but will eventually be integrated into the municipal museum of Shanshan, Xinjiang Autonomous Province that is currently under construction. All specimens have been assigned a combined PMOL-Sino-German Cooperation Project (SGP) number, which will be deposited with the specimens once the museum in Shanshan is operational. The detailed coordinates of the locality are archived at PMOL and will be disclosed to qualified researchers interested in studying the site.

Specimen PMOL-SGP A0100-1 was discovered with the carapace exposed in dorsal view in 2009 below a small cliff and was cut out of the hard sandstone ledge in a block with an ICS diamond chain rock saw in 2011. Subsequent preparation revealed that the slab contained two more individuals with PMOL-SGP A0100-2 cut in half through the long axis during excavation. The slab in total includes PMOL-SGP A0100-3: shell with carapace partially exposed, femora, skull and lower jaw; PMOL-SGP A0100-2: shell (plastron not exposed), partial neck, left foot and left hand and PMOL-SGP A0100-1: posteriorly incomplete carapace, neck, crushed skull with articulated mandible, left and right hand and incomplete left posterior limb.

The anatomy of fossil taxa was reviewed mostly based on personal observations of published material and with the help of photographs. The following fossil taxa were studied first hand: *Allopleuron hoffmanni* (Gray, 1831) [32] (NHMUK R42913); *Chubutemys copelloi* Gaffney et al., 2007 [10] (MPEF-PV1236); *Dracochelys bicuspis* Gaffney and Ye, 1992 [33] (IVPP V4075); *Hangaiemys hoburensis* Sukhanov and Narmandakh, 1974 [34] (PIN 3334-4, PIN 3334-34, PIN 3334-35, PIN 3334-36, PIN 3334-37); *Heckerochelys romani* Sukhanov, 2006 [35] (PIN 4561-2 and PIN 4719-34); *Hoyasemys jimenezi* Pérez-García et al., 2012 [36] (MCCM-LH-84); *Helochelydra nopcsai* Lapparent de Broin and Murelaga, 1999 [37] (IWCMS 1998.21); *Judithemys sukhanovi* Parham and Hutchison, 2003 [9] (TMP 87.2.1); *Kallokibotion bajazidi* Nopcsa, 1923 [38] (NHMUK R4921 and NHMUK R4925); *Kayentachelys aprix* Gaffney et al., 1987 [39] (MNA V1558, MCZ 8917); *Macrobaena mongolica* Tatarinov, 1959 [40] (PIN 533-4); *Manchurochelys manchoukuoensis* Endo and Shikama, 1942 [41] (PMOL AR00008); *Meiolania platyceps* Owen, 1886 [42] (NHMUK R682); *Mongolemys elegans* Khosatzky and Mlynarski, 1971 [43] (five uncatalogued skulls at the collections of PIN); *Mongolochelys efremovi* Khozatsky, 1997 [44] (PIN 552-459 and two uncatalogued skulls); *Naomichelys speciosa* Hay, 1908 [45] (FMNH PR 273); *Niolamia argentina* Ameghino 1899 [46]*Notoemys laticentralis* Cattoi and Freiberg, 1961 [47] (cast of MOZP 2487); *Odontochelys semitestacea* Li et al., 2008 [48] (IVPP V13240); *Ordosemys leios* Brinkman and Peng, 1993 [49] (IVPP V9534-1); *Peligrochelys walshae* Sterli and de la Fuente, In press [16] (MACN PV CH 2017, MACN PV CH 2017); *Portlandemys mcdowelli* Gaffney, 1975 [50] (NHMUK R2914, NHMUK R3163, NHMUK R3164); *Proganochelys quenstedti* Baur, 1887 [51] (SMNS 16980); *Rhinochelys elegans* Lydekker, 1889 [52] (NHMUK R27); *Sandownia harrisi* Meylan et al., 2000 [53] (MIWG 3480); *Sinemys gamera* Brinkman and Peng, 1993 [54] (IVPP V9532-11); *Sinemys brevispinus* Tong and Brinkman, In press [55] (IVPP V9538-1); *Solnhofia parsonsi* Gaffney, 1975 [56] (TM 4023); *Toxochelys latiremis* Cope, 1873 [57] (NHMUK R4530 and NHMUK R3902); *Xinjiangchelys* (*Annemys*) *levensis* Sukhanov and Narmandakh, 2006 [58] (PIN 4636-4-2, [59]); *Xinjiangchelys* (*Annemys*) *latiens* Sukhanov and Narmandakh, 2006 (PIN 4636-6-2, [59]); and *Xinjiangchelys radiplicatoides* Brinkman et al., 2013 [15] (IVPP V18104).

The following taxa were studied on the basis of photographs: *Adocus lineolatus* Cope, 1874 [60] (CCM 60-15); *Basilochelys macrobios* Tong et al., 2009 [61] (MD 8-2); *Bouliachelys suteri* Kear and Lee, 2006 [62] (SAM P41106); *Meiolania platyceps* AM F: 18671; *Plesiochelys etalloni* Pictet and Humbert, 1857 [63] (MH 435); *Pleurosternon bullockii* Owen 1842 [64] (UMZC T1041).

### Osteological terminology

**Figure 3:**
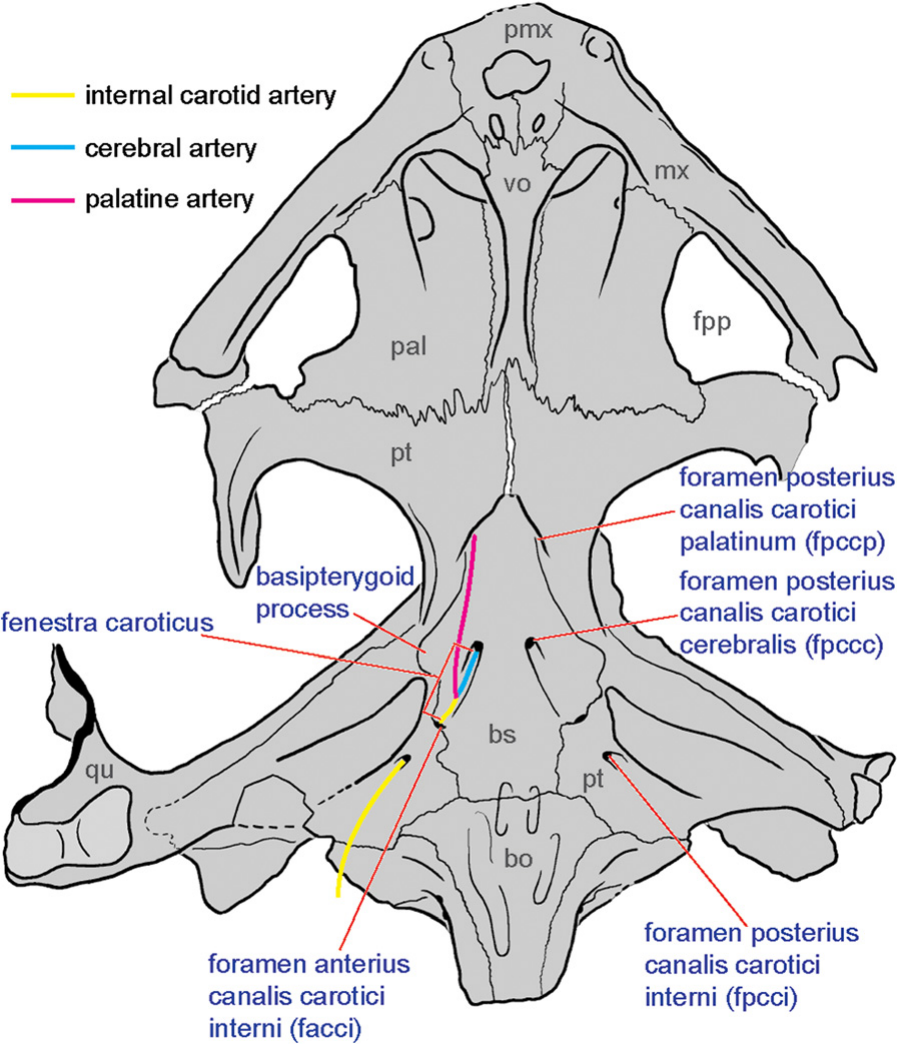
**Proposed internally consistent nomenclature for the osseous portion of the carotid circulation system of turtles as exemplified on the skull of *Dracochelys bicuspis *(IVPP V4075).** Abbreviations: **bo**: basioccipital, **bs**: basisphenoid, **fpp**: foramen palatinum posterius, **mx**: maxilla, **pal**: palatine, **pmx**: premaxilla, **pt**: pterygoid, **qu**: quadrate, **vo**: vomer.

The cranial nomenclature presented by Gaffney [65,66] has been highly influential, because all anatomical systems of the cranium were clearly described and illustrated in these publications and because a broad audience was thereby enabled to apply these names consistently to the skulls of fossil and recent turtles. Only in the last few years have some shortcomings become apparent, however, particularly in regards to the nomenclature of the carotid system and we herein seek to rectify this situation by providing an internally consistent nomenclatural system for this anatomical region (Figure 3).

The internal carotid artery of most turtles, like most amniotes, splits into a cerebral and a palatine (lateral) branch. Although these structures are interrelated, they can be thought of as three different vessels, which are herein terms the internal carotid artery, the cerebral artery, and the palatine artery. New insights into the cranial anatomy of basal turtles [15,20,67] has revealed that these three blood vessels can enter the skull through three non-homologous foramina and that they can also exit the skull through three non-homologous foramina, for a total of six non-homologous foramina. The nomenclatural system of Gaffney [65,66] proved to be confusing, because it only provides three names for these six foramina (i.e., foramen *anterior* [italics added for emphasis] canalis carotici interni, foramen *posterior* canalis carotici interni, and foramen caroticum laterale) and because these names were defined as applying to inappropriate portions of the carotid system. For instance, the foramen *anterior* canalis carotici interni was defined as applying to the exit of the cerebral artery, not to the exit of the internal carotid artery, whereas the foramen posterior canalis *carotici interni* could either be the entry of the internal carotid artery or of the cerebral artery [65,66]. An addition oddity of this nomenclatural system that makes it difficult for neophytes to learn that the palatine artery is situated in the “lateral canal,” not the palatine canal. Incidentally, the use of the “-ior” suffix (as in *anterior* and *posterior*) is not appropriate, given that the “-ius” suffix is the proper neuter singular ending in Latin.

Sterli et al. [20] were the first to realize these deficiencies in the nomenclatural system of Gaffney [65,66] and proposed new terms, but these new terms are not sufficient to name all six potential foramina and they break with tradition set by Gaffney [65,66] in their grammatical construction. These inconsistencies were partially addressed recently [15] but some parts of the system still remain unnamed and the palatine artery is still defined as sitting in the lateral canal.

We herein propose a new nomenclatural system that attempts to follow the grammatical precedence set forth by Gaffney [65,66], but that breaks tradition by providing names for all potential foramina and by renaming the lateral canal the palatine canal. This nomenclatural system consists of a total of 10 new terms (Figure 3):

#### ***Canalis caroticus internus***

The bony canal that holds any portion of the internal carotid artery, absent, among others, in basal turtles and paracryptodires.

#### ***Foramen posterius canalis carotici interni (fpcci)***

The posterior entry of the internal carotid artery, absent, among others, in basal turtles and paracryptodires.

#### ***Foramen anterius canalis carotici interni (facci)***

The anterior exit of the internal carotid artery, only present in turtles with a fenestra caroticus.

#### ***Canalis caroticus cerebralis***

The bony canal that holds any portion of the cerebral artery, present in all turtles.

#### ***Foramen posterius canalis carotici cerebralis (fpccc)***

The posterior entry of the cerebral artery, not developed in turtles where the split of the internal carotid artery into the cerebral and palatine branches is covered by bone.

#### ***Foramen anterius canalis carotici cerebralis (faccc)***

The anterior exit of the cerebral artery, present in all turtles, typically located near the dorsum sellae.

#### ***Canalis caroticus palatinum***

The bony canal that holds any portion of the palatine artery, generally absent in turtles with an open interpterygoid vacuity.

#### ***Foramen posterius canalis carotici palatinum (fpccp)***

The posterior entry of the palatine artery, generally developed in turtles with a closed interpterygoid vacuity, but not in those where the split of the internal carotid artery into the cerebral and palatine branches is covered by bone.

#### ***Foramen anterius canalis carotici palatinum (faccp)***

The anterior exit of the palatine artery, generally present in turtles with a close interpterygoid vacuity.

#### ***Fenestra caroticus (fca)***

A figurative bony window into the otherwise closed carotid system, which exposes the split of the internal carotid artery into the cerebral and palatine branches. The window is posteriorly defined by the foramen anterius canalis carotici interni and anteriorly defined by the foramen posterius canalis carotici cerebralis and the foramen posterius canalis carotici palatinum or the interpterygoid vacuity.

### Phylogenetic analysis

A phylogenetic analysis was performed using TNT [68,69] using a modified version of a previous character/taxon matrix [16], which in return is based on earlier studies [3,5,59,70] [Additional file 1]. Part of the changes are reported in an in press paper by Rabi et al. [59] and these are repeated below for the sake of clarity. Five taxa were added to the matrix [16], including *Xinjiangchelys radiplicatoides*, *X. junggarensis* (sensu Brinkman et al. 2008 [71]), *X*. (*Annemys*) *levensis*, *X*. (*Annemys*) *latiens*, and *Basilochelys macrobios*. The scorings of *X. radiplicatoides* are primarily based on the literature [15], those of *X*. *junggarensis* (=*X*. *latimarginalis*[72]) on personal observation of IVPP material from Pingfengshan [72], those of *X*. (*Annemys*) *levensis*, and *X*. (*Annemys*) *latiens* based on personal observation of PIN material, and those of *B*. *macrobios* based on the literature [60] and photographs obtained from H. Tong. The following scorings were changed relative to the original matrix [16] (the earlier scorings are in parenthesis): Epiplastron B: *Hangaiemys hoburensis*: 1 (?), *Sinemys lens* Wiman, 1930 [73] 1 (?); Pterygoid B: *H. hoburensis* 1 (2), *Dracochelys bicuspis* 1 (2), *Pleurosternon bullockii* 1 (2), *Kallokibotion bajazidi* 1 (2), *Mongolochelys efremovi* 1 (2), *Chubutemys copelloi* 1 (2), *Eileanchelys waldmani* Anquetin, 2009 [74] ? (2); Carapace D: *H. hoburensis* 0 (?), *Chengyuchelys baenoides* Young and Chow, 1953 [75] (IVPP-V6507) 0 (1); Carapace E: *H. hoburensis*-(?); Vertebral A: *Siamochelys peninsularis* Tong et al., 2002 [76] ? (1); Vertebral C: *S. peninsularis* ? (1); Anal A: *S. peninsularis* ? (0), *Ch. baenoides* ? (0); Entoplastron B: *Ch. baenoides* ? (1); Mesoplastron A: *S. peninsularis* 2 (0); Hypoplastron A: *Ch. baenoides* ? (0); Xiphiplastron A-B: *Ch. baenoides* ? (0); Dorsal Rib A: *S. peninsularis* ? (2); Plastral Scute B: *S. peninsularis* 1 (0).

Further modifications relative to Rabi et al. in press [59] include the addition of *Xinjiangchelys wusu* to the matrix and changing of the following scorings: Supraoccipital A: *X*. (*Annemys*) *levensis* 1 (0); *X*. *radiplicatoides* ? (0); *X*. (*Annemys*) *latiens* ? (0); Pterygoid B: *Sphenodon punctatus* 0 (2), *Anthodon serrarius* 1 (2), *Peligrochelys walshae* 2 (1), *Niolamia argentina* 2 (?); Dentary A: *X*. (*Annemys*) *levensis* 0 (1); *X*. *junggarensis* ? (1).

The character Cervical Vertebrae A was omitted from the analysis because we found it difficult to replicate this character objectively and perceived a number of inconsistencies in the matrix [59]. The character Diploid Number A was also omitted following previous studies [3,59,77].

The following characters were treated as ordered: 7 (Nasal A), 19 (Parietal H), 27 (Squamosal C), 40 (Maxilla D), 42 (Vomer A), 50 (Quadrate B + C), 52 (Antrum Postoticum A), 59 (Pterygoid B), 81 (Opisthotic C), 82 (Opisthotic D), 89 (Stapedial Artery B), 98 (Canalis Caroticum F), 120 (Carapace A), 121 (Carapace B), 130 (Peripheral A), 133 (Costal B), 138 (Supramarginal A), 158 (Hyoplastron B), 159 (Mesoplastron A), 161 (Hyoplastron B), 176 (Abdominal A), 213 (Cleithrum A), 214 (Scapula A), 232 (Manus B), 233 (Manus C). *Sphenodon punctatus, Owenetta kitchingorum, Simosaurus gaillardoti* and *Anthodon serrarius* were designated as outgroups [16,59]. Although, there is growing evidence for a turtle-archosaur clade among molecular studies, morphological analyses still suggest lepidosaurian or parareptilian affinities for turtles at the moment. As it turns out, however, the choice of outgroup is irrelevant, as all outgroups reveal that the presence of teeth and the lack of a complete shell should be considered primitive for turtles and that the partially shelled, toothed taxon *Odontochelys semitestacea* is therefore sister to all turtles. The fusion of the basicranium discussed in our paper occurs far deeper within the turtle tree and is therefore not influenced by the choice of outgroups, but rather by the arrangement of basal turtles.

Given that this analysis is focused on the phylogenetic relationships and placement of xinjiangchelyid turtles, we decided to crop taxa not pertinent to these questions (e.g., most derived baenids, most meiolaniforms) and a broad spectrum of taxa known from fragmentary material only (see Appendix A for a complete list) in order to reduce the size of the matrix [59]. The resulting matrix consists of 237 characters for a total of 84 terminal taxa. The character-taxon matrix and the tnt. file are found under [Additional files 1, and 2], respectively.

The relationships of living cryptodiran taxa were manually constrained according to recent results of molecular phylogenetic studies (following previous studies [1,2,59]), without assuming a priori, however, that Trionychia nests within Cryptodira [78,79]. The internal relationships of durocryptodires were constrained using a molecular topology [79] (i.e., (Emydidae (Geoemydidae + Testudinidae)) + (Chelonioidea (Chelydridae + Kinosternoidea))). The complete list of taxa designated as floaters can be found in Appendix B. A first run of heuristic search tree-bisection-reconnection, using thousands of random addition sequence replicates and 10 trees saved per replicate, failed to find all the most parsimonious trees (MPT) and therefore the heuristic search was repeated until the MPTs were found 30 times during each replicate (using the command “xmult = hits 30;”). The trees retained in the memory were exposed to a second round of tree-bisection-reconnection.

### Systematic paleontology

TESTUDINATA Klein, 1760 [80]

TESTUDINES Batsch, 1788 [81]

XINJIANGCHELYIDAE Nessov in Kaznyshkin et al., 1990 [7] (sensu Rabi et al., In press [59])

#### ***Remark***

We follow the phylogenetic definition of Xinjiangchelyidae used in Rabi et al. (In press [59]) where Xinjiangchelyidae is defined as the most inclusive clade containing *Xinjiangchelys junggarensis* Ye, 1986 [82], but not *Sinemys lens*, *Macrobaena mongolica*, or any species of Recent turtle.

*Xinjiangchelys* Ye, 1986 [82]

*Remark:* A number of genera other than *Xinjiangchelys* have been referred to Xinjiangchelyidae in recent years, including *Chengyuchelys* Young and Chow, 1953 [75]; *Tienfuchelys* Young and Chow, 1953 [75]; *Annemys* Sukhanov and Narmandakh, 2006 [58]; *Shartegemys*, Sukhanov and Narmandakh, 2006 [58]; *Yanduchelys* Peng et al. 2005 [83]; *Protoxinjiangchelys* Tong et al. 2012 [13] ([8,13-15,58,71]). The majority of these genera are sufficiently diagnosed relative to *Xinjiangchelys*, but there is no up-dated diagnosis available for *Xinjiangchelys*. This taxon has therefore been rendered a waste-backed taxon defined by what it is *not*. To avoid further complications we suggest using a more inclusive definition for *Xinjiangchelys* that includes all species of Xinjiangchelyidae (sensu Rabi et al., In press [59]) until the phylogenetic relationships of the included taxa can be determined more confidently.

**Figure 4:**
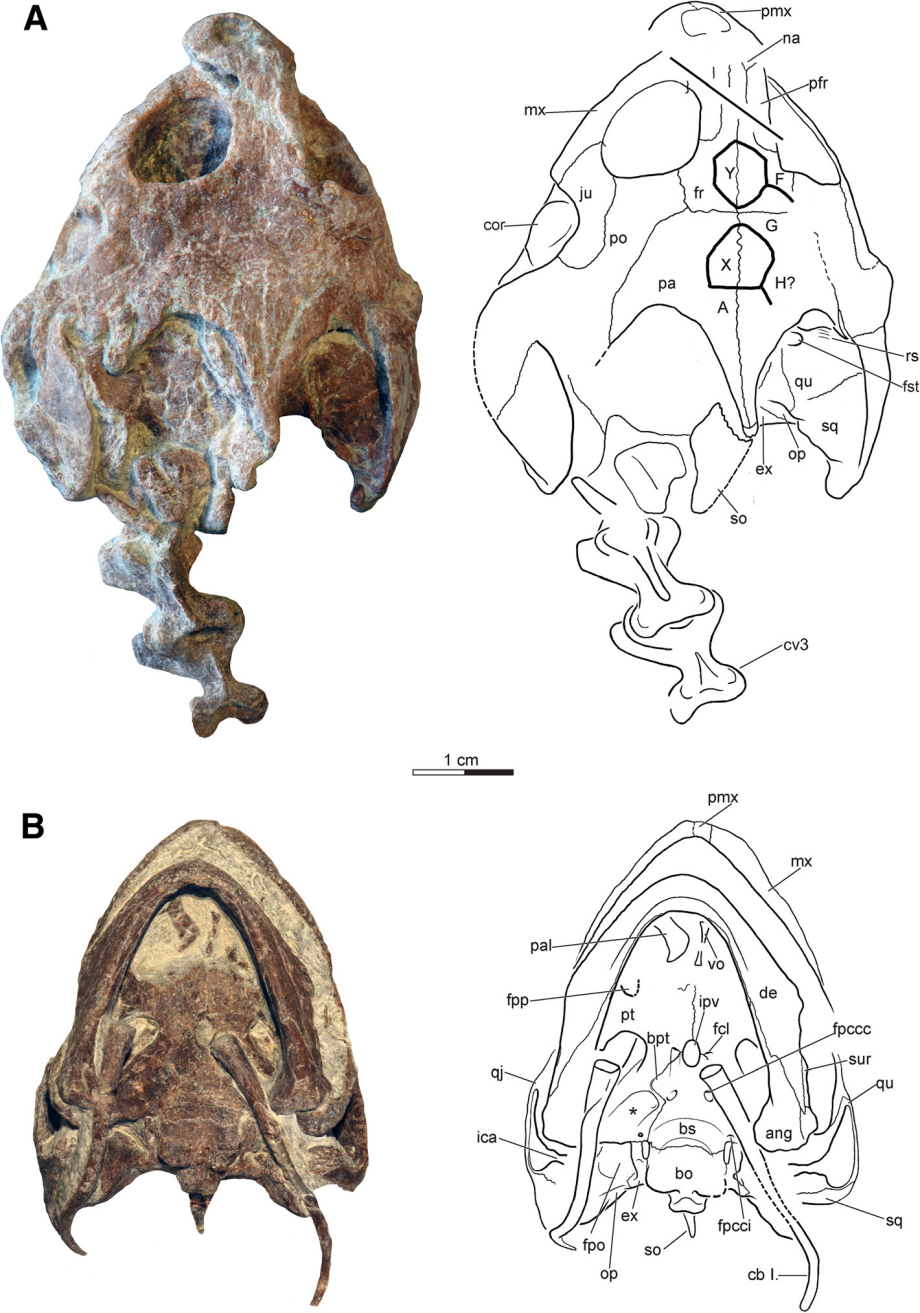
**Skulls and partial neck of *Xinjiangchelys wusu*, Middle Jurassic, ?Qigu Formation, “Turtle Cliff”, Shanshan area, Turpan Basin, Xinjiang Autonomous Province, China. A**, PMOL-SGP A0100-1 (holotype), photograph and line drawing of skull and anterior cervical vertebrae in dorsal view; **B**, PMOL-SGP A0100-3, photograph and line drawing of skull, mandible and hyoid apparatus in ventral view. Abbreviations: **ang**: angular, **bo**: basioccipital, **bpt**: basipterygoid process, **bs**: basisphenoid, **cb I.**: cornu branchiale I, **cor**: coronoid, **cv**: cervical vertebra, **de**: dentary, **ex**: exoccipital, **fcl**: foramen caroticum laterale, **fpccc**: foramen posterius canalis carotici cerebralis, **fpcci**: foramen posterius canalis carotici interni, **fpo**: fenestra postotica, **fpp**: foramen palatinum posterius, **fr**: frontal,** fst**: foramen stapedio-temporale, **ica**: incisura columella auris, **ipv**: interpterygoid vacuity, **ju**: jugal, **mx**: maxilla, **na**: nasal, **op**: opisthotic, **pa**: parietal, **pal**: palatine, **pfr**: prefrontal, **pmx**: premaxilla, **po**: postorbital, **pt**: pterygoid, **qj**: quadratojugal, **qu**: quadrate, **rs**: rugose surface of processus trochlearis oticum, **so**: supraoccipital, **sq**: squamosal, **sur**: surangular, **vo**: vomer, * refers to fossa pterygoidea. A, X, G, H, Y refer to scales after Sterli and de la Fuente [16].

**Figure 5:**
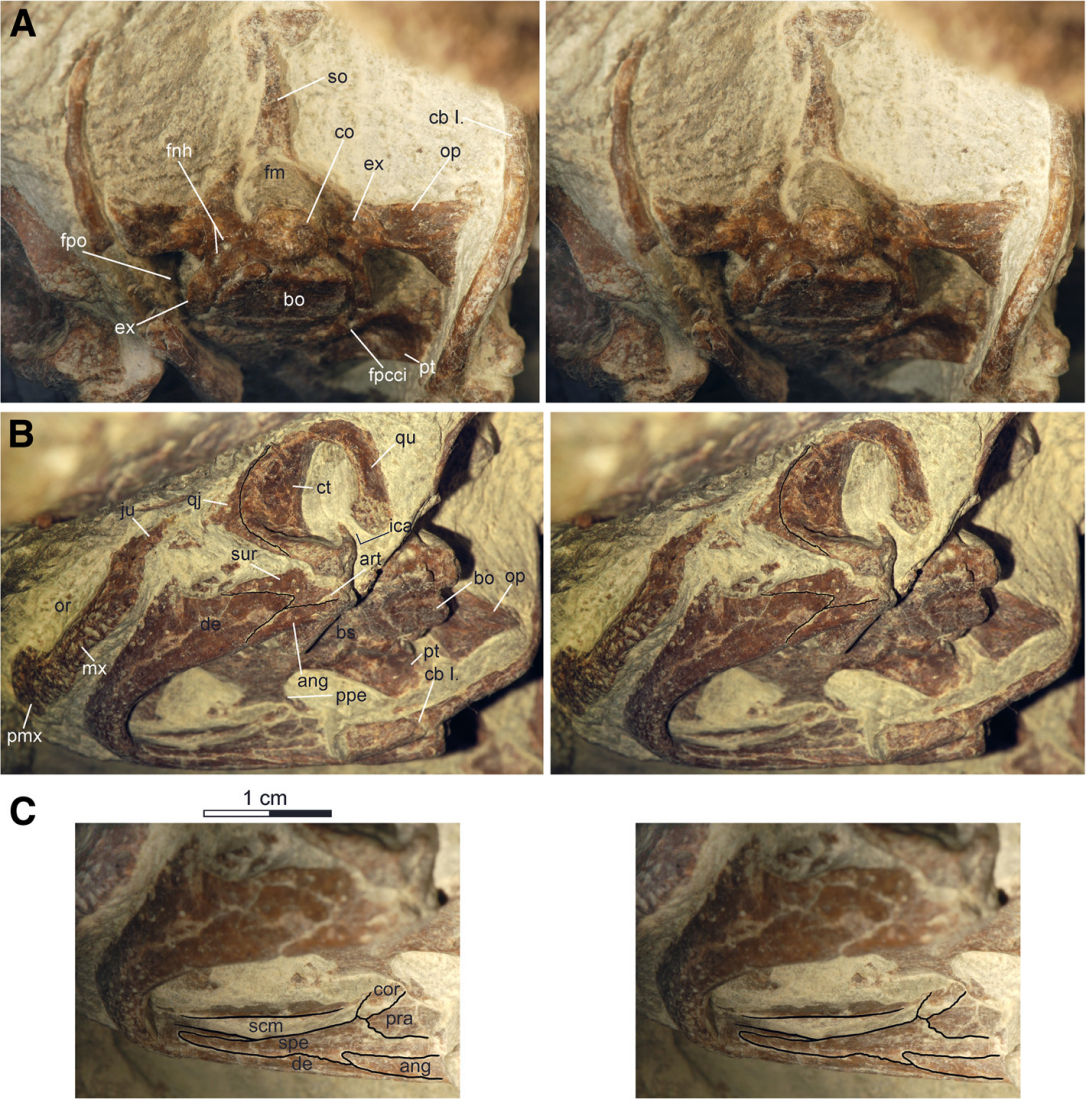
**PMOL-SGP A0100-3, skull, mandible and hyoid apparatus of *Xinjiangchelys wusu*, Middle Jurassic, ?Qigu Formation, “Turtle Cliff”, Shanshan area, Turpan Basin, Xinjiang Autonomous Province, China. A**, occipital view of skull; **B**, left ventrolateral view of skull and mandible; **C**, medial view of right ramus of mandible. Abbreviations: **ang**: angular, **art**: articular, **bo**: basioccipital, **bs**: basisphenoid, **cb I.**: cornu branchiale I., **co**: condylus occipitalis, **cor**: coronoid, **ct**: cavum tympani, **de**: dentary, **ex**: exoccipital, **fm**: foramen magnum; **fnh**: foramen nervi hypoglossi, **fpcci**: foramen posterius canalis carotici interni, **fpo**: fenestra postotica, **ica**: incisura columella auris, **ju**: jugal, **mx**: maxilla, **op**: opisthotic, **or**: orbit, pmx: premaxilla, **ppe**: processus pterygoideus externus, **pra**: prearticular, **pt**: pterygoid, **qj**: quadratojugal, **qu**: quadrate, **scm**: sulcus cartilaginis Meckelii, **so**: supraoccipital, **spe**: splenial, **sur**: surangular.

**Figure 6:**
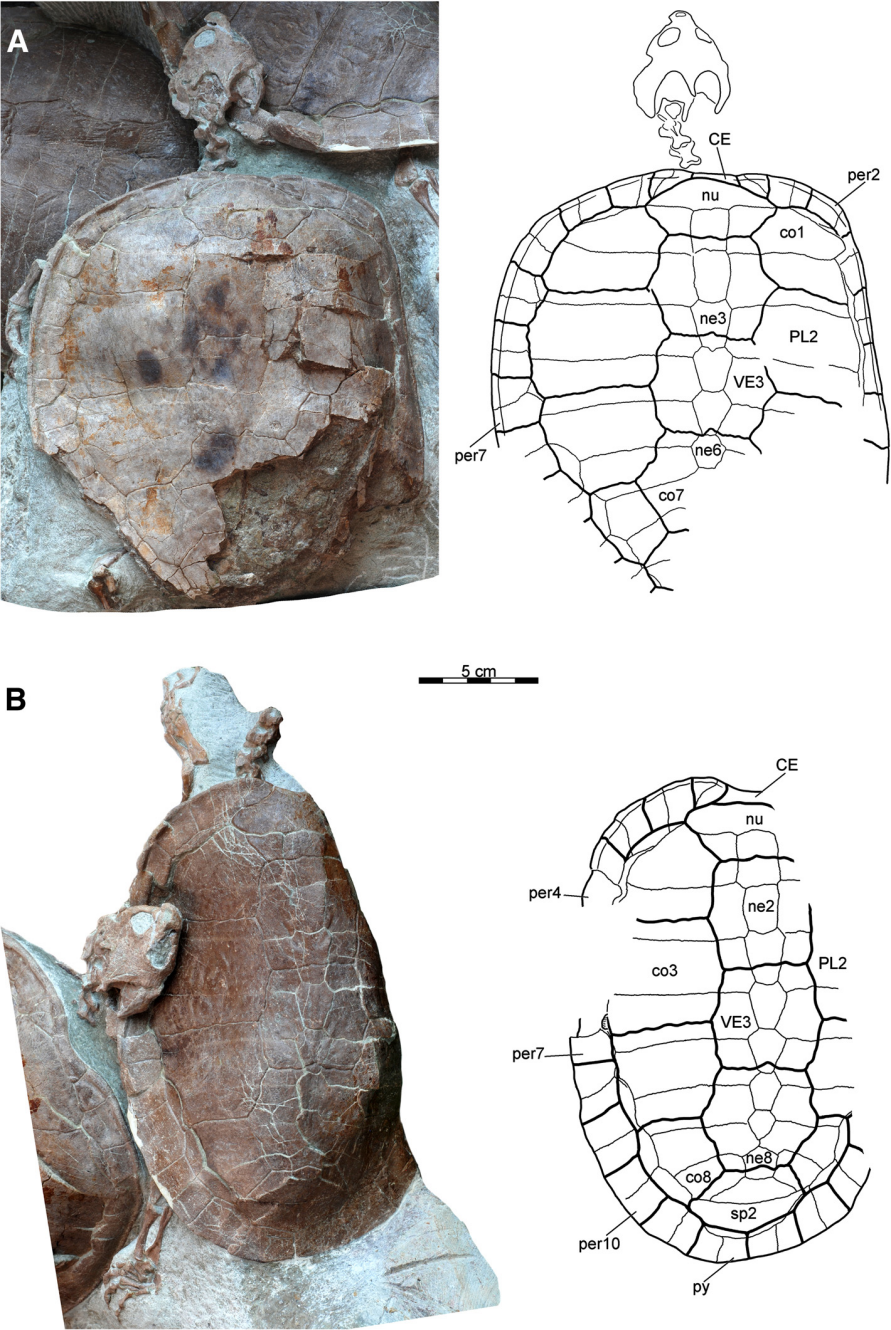
**Carapaces of *Xinjiangchelys wusu*, Middle Jurassic, ?Qigu Formation, “Turtle Cliff”, Shanshan area, Turpan Basin, Xinjiang Autonomous Province, China. A**, PMOL-SGP A0100-1 (holotype), photograph and line drawing; **B**, PMOL-SGP A0100-2, photograph and line drawing. Abbreviations: **CE**: cervical scute, **co**: costal, **ne**: neural, **nu**: nuchal, **per**: peripheral, **PL**: pleural, **VE**: vertebral, **py**: pygal, **sp**: suprapygal.

**Figure 7:**
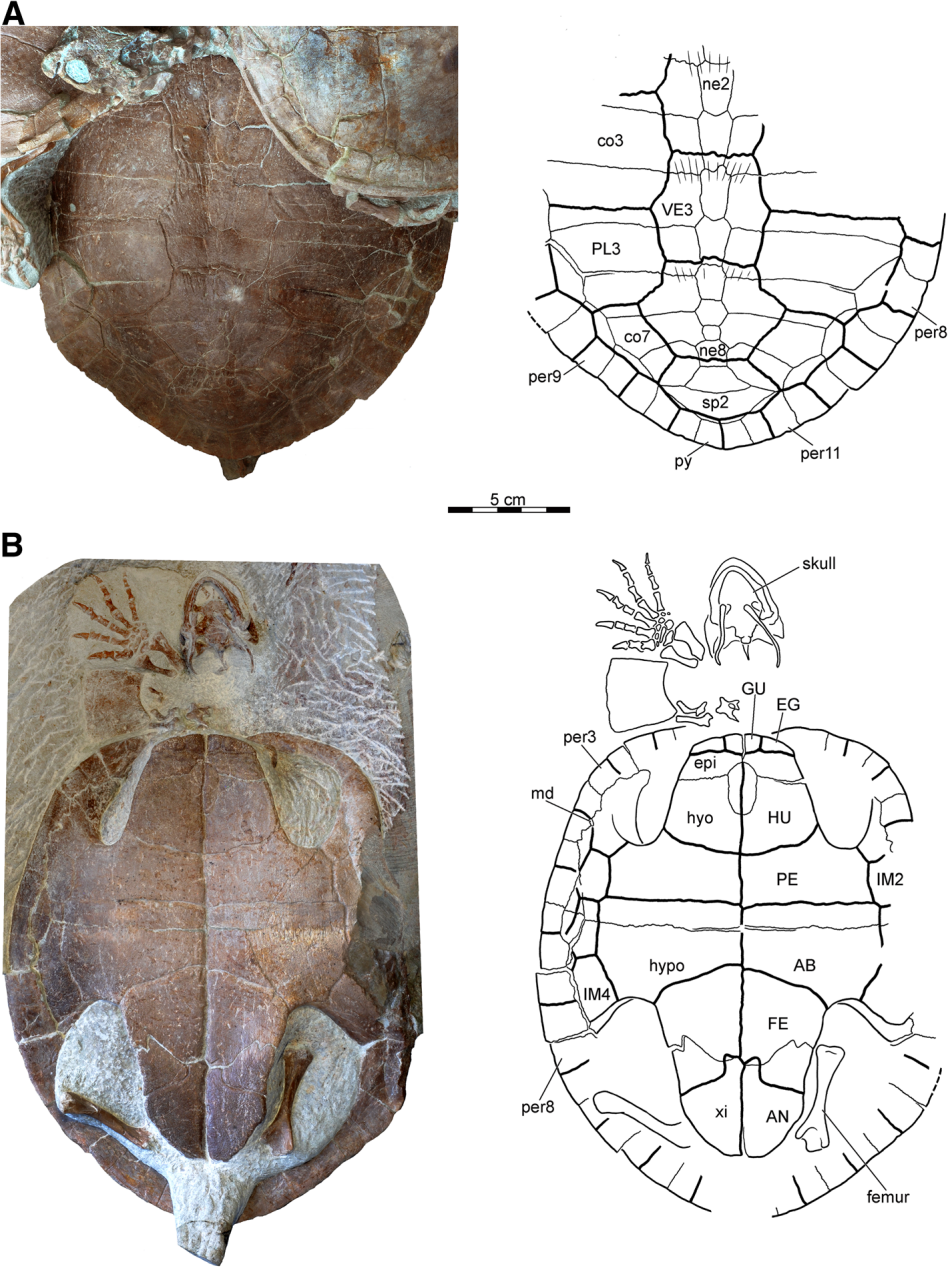
**PMOL-SGP A0100-3 *Xinjiangchelys wusu *shell, Middle Jurassic, ?Qigu Formation, “Turtle Cliff”, Shanshan area, Turpan Basin, Xinjiang Autonomous Province, China. A**, photograph and line drawing of posterior two third of carapace; **B**, photograph and line drawing of plastron. The right forelimb in 'B’ does not belong to PMOL-SGP A0100-3 but to PMOL-SGP A0100-1. Abbreviations: **AB**: abdominal, **AN**: anal, **co**: costal, **EG**: extra gular, **epi**: epiplastron,** FE**: femoral, **GU:** gular, **HU**: humeral, **hyo**: hyoplastron, **hypo**: hypoplastron, **IM**: inframarginal, **md**: musk duct foramen, **ne**: neural, **per**: peripheral, **PE**: pectoral, **PL**: pleural, **py**: pygal, **sp**: suprapygal, **VE**: vertebral, **xi**: xiphiplastron.

**Figure 8:**
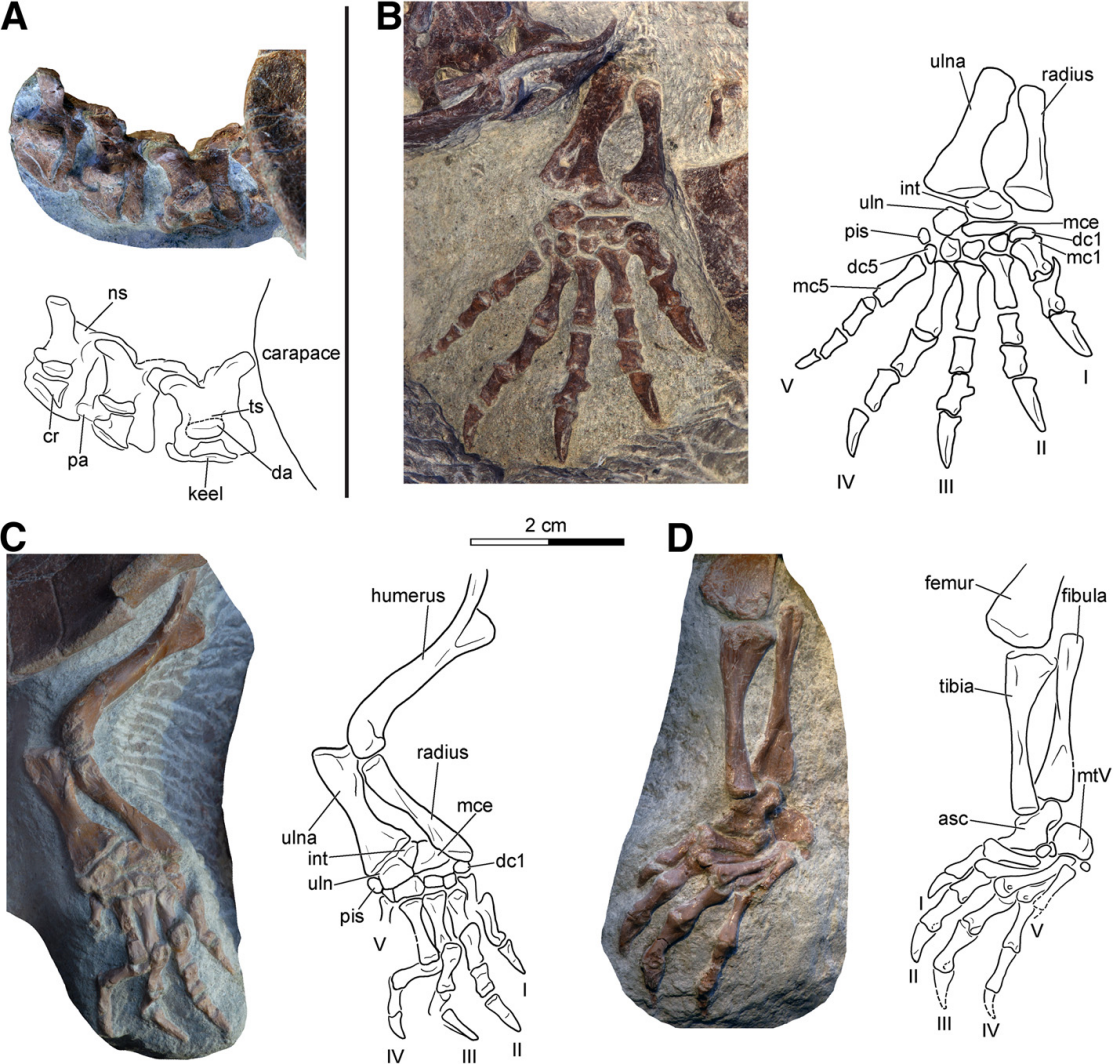
**Neck and appendicular elements of *Xinjiangchelys wusu*, Middle Jurassic, ?Qigu Formation, “Turtle Cliff”, Shanshan area, Turpan Basin, Xinjiang Autonomous Province, China. A**, PMOL-SGP A0100-2, photograph and line drawing of articulated cervical vertebrae III-V in lateral view; **B**, PMOL-SGP A0100-1, photograph and line drawing of right distal fore limb; **C**, PMOL-SGP A0100-2, photograph and line drawing of left fore limb; **D**, PMOL-SGP A0100-2, photograph and line drawing of left hind limb. Abbreviations: **asc**: fused astragalocalcaneum, **cr**: cervical rib, **da**: diapophysis, **dc**: distal carpal, **int**: intermedium, **mc**: metacarpal, **mce**: medial central, **mt**: metatarsal, **ns**: neural spine, **pa**: parapophysis, **pis**: pisiform, **ts**: transverse process, **uln**: ulnare.

*Xinjiangchelys wusu* sp. nov.

(Figure 2, Figures 4, 5, 6, 7, and 8)urn:lsid:zoobank.org:pub:2BCCC095-7622-4 F27-8 F80-6199F24690B5

#### ***Holotype***

PMOL-SGP A0100-1, a partial skeleton, including the skull exposed in dorsal view (Figures 2, 4, 6 and 8B).

#### ***Referred material***

PMOL-SGP A0100-3, partial skeleton (Figures 2, 4, 5 and 7,); PMOL-SGP A0100-2, partial skeleton without skull, plastron not exposed (Figures 2, 6 and 7A, C-D).

#### ***Locality and horizon***

Turtle Cliff Fossil Locality (see Geological Settings), Shanshan, Turpan Basin, Xinjiang Autonomous Province, People’s Republic of China (Figure 1); ?Qigu Formation, Upper Jurassic.

#### ***Etymology***

*wusu* refers to a small town in Xinjiang Autonomous Province.

#### ***Diagnosis***

A species of *Xinjiangchelys*; skull differing from *X.* (*Annemys*) *levensis* in the prefrontals being fully separated by the frontals; from *X*. (*Annemys*) *latiens* by the broader skull and the extensive jugal and frontal contribution to the orbit, from *X. radiplicatoides* by the flattened skull and the presence of a remnant of the interpterygoid vacuity. Shell differing from *X*. *chowi* Matzke et al. [84]*X*. *qiguensis* Matzke et al., [85]*X*. *tianshanensis* Kaznyshkin et al. [7] and *X*. *junggarensis* (sensu Brinkman et al. 2008 [70]) by the narrow vertebral scales.

### Description

#### ***Skull***

##### 

**Preservation** The skull of PMOL-SGP A0100-1 is exposed only in dorsal and lateral views, whereas its palatal side is covered by the carapace of PMOL-SGP A0100-2 (Figure 2). It is dorsoventrally crushed and the preorbital region is slightly shifted from its original position. The skull of PMOL-SGP A0100-3, on the other hand, is exposed in ventral view and in articulation with the hyoids and the mandible (Figures 4B and 5).

##### 

**Scales** Some of the cranial scales are traceable in PMOL-SGP A0100-1, but most of them are not apparent (Figure 4A). Using a recently suggested nomenclatural system [16] we identify the unpaired scale Y on the posterior half of the frontal posteriorly bordered by the paired scale F. Scale G is bordered by the unpaired parietal scale X posteriorly. Scale A is another unpaired scale of the parietal found posteriorly to scale X. Scale H may have also been present laterally to scale X. The skull roof is otherwise decorated with fine grooves and very shallow pits that do not show a clear pattern.

##### 

**Nasals** The nasals are very poorly preserved but their sutures with the frontal and the prefrontal are partially traceable on the right side of PMOL-SGP A0100-1 (Figure 4A). They are reduced, posteriorly tapering elements that are partially separated by the anterior frontal process. The nasals contribute to the formation of the external nares.

##### 

**Prefrontals** The dorsal plate of the prefrontals is elongate and medially separated from its counterpart by the anterior frontal process (Figure 4A). The prefrontal contacts the nasal anteriorly and the maxilla ventrally. The descending process of the prefrontal has a wide contact with the palatines within the fossa orbitalis. Its contact with the vomer is not visible, but given the large size of the prefrontal pillars it was very likely present. The frontal forms the anterior half of the dorsal margin of the orbit.

##### 

**Frontals** The frontals form an anterior process that is wedged between the prefrontals (Figure 4A). The posterior half of the dorsal margin of the orbit is formed by the frontals. The orbit has a subcircular outline and faces dorsolaterally.

##### 

**Parietals** The dorsal plate of the parietals exhibits a relatively deep temporal emargination that reaches beyond the level of the anterior border of the cavum tympani (Figure 4A). The parietal meets the frontal anteriorly and has a long contact with the postorbital. Even though the parietals are slightly shifted from their original position in PMOL-SGP A0100-1, their posterolateral tips also touched the squamosal, as seen on the right side. Dorsoventral crushing obscures the structures of the processus inferior parietalis.

##### 

**Jugal** The jugal area is compressed and its entire lateral surface is exposed in dorsal view in PMOL-SGP A0100-1 and it is also partially visible in PMOL-SGP A0100-3 (Figures 4A and 5B). It sends a long posterior process along the postorbital but it is unclear whether it meets the quadratojugal. The skull exhibits a moderate cheek emargination that exposes the coronoid process of the mandible. Anteriorly, the jugal forms the posterolateral margin of the orbit and contacts the maxilla. It is unclear whether the ventral plate of the jugal contacts the posterior end of the triturating surface and/or the anterolateral tip of the external pterygoid process.

##### 

**Quadratojugal** The quadratojugal is a reduced, flat element that is best preserved in PMOL-SGP A0100-3, though not fully exposed (Figure 5B). It has no clear contact with the jugal anteriorly but this is all but certain. Dorsally, it meets the postorbital and posteriorly it borders the cavum tympani. Its lower rim of the skull is emarginated, which gives the quadratojugal a subtriangular outline. The quadratojugal sends a pair of narrow and tapering processes along the dorsal and the ventral margins of the cavum tympani, respectively. The dorsal one of these processes is wedged between the postorbital and the quadrate and appears not to reach the squamosal. The ventral one terminates slightly before the level of the condylus mandibularis.

##### 

**Squamosal** The squamosal is better preserved on the right side of PMOL-SGP A0100-1, the left one being compressed and the lateral plate being exposed when the skull is viewed dorsally (Figure 4). The lateral surface of the squamosal is smooth and there is no squamosal horn. The squamosal has a very short point-like contact with the parietal along the anterior margin of the upper temporal emargination. There is no contribution to the formation of the anterior opening of the antrum postoticum as seen in PMOL-SGP A0100-3. Medially, the squamosal contacts the quadrate and may even have a short contact with the opisthotic within the upper temporal fossa. The contact of the ventral portion with the opisthotic and the quadrate is not exposed in either specimen.

##### 

**Postorbital** The postorbitals are long elements; they form the posterodorsal margin of the orbit and also contribute to the rim of the upper temporal emargination (Figure 4A). The postorbital has an anteroventral contact with the jugal, a posteroventral contact with the quadratojugal, a long lateral contact with the parietal, and also meets the squamosal posteriorly.

##### 

**Premaxilla** The premaxillary region is shifted anteriorly from the original position and damaged in PMOL-SGP A0100-1 (Figure 4). The premaxilla forms the ventral margin of the external nares and contacts the other premaxilla medially and the maxilla posterolaterally. The external nares are undivided. Only little of the ventral aspect of the premaxillary region is exposed but it is apparent that there is no premaxillary hook.

##### 

**Maxilla** The maxilla forms the ventral margin of the orbit, sends a dorsal process to contact the descending pillar of the prefrontal, and contacts the premaxilla anteriorly and the jugal posteriorly (Figures 4 and 5B). The triturating surface is only partially exposed but it is apparently narrow and straight with a sharp and low labial ridge.

##### 

**Vomer** A single, slightly damaged and displaced, elegant vomer is present in PMOL-SGP A0100-3 exposed in dorsal view (Figure 4B). Its outline is very similar to that of *Xinjiangchelys levensis*.

##### 

**Palatine** The right palatine is preserved incompletely and shifted from the original position in PMOL-SGP A0100-3 (Figure 4B). It shows an extensive free lateral margin that is indicative of a large foramen palatinum posterius.

##### 

**Quadrate** Apart from the region of the cavum tympani, the right quadrate of PMOL-SGP A0100-1 is in good condition whereas the left otic region is badly fragmented and compressed (Figures 4 and 5B). In PMOL-SGP A0100-3 the region of the cavum tympani is exposed in lateral view. The cavum tympani is anteriorly bordered by the quadratojugal and by the squamosal dorsally and posteriorly. The incisura columella auris is an open but tight notch and there is no precolumellar fossa. The antrum postoticum is well developed and its opening is formed entirely by the quadrate, although the squamosal comes very close to the lateral rim. The quadrate contacts within the upper temporal fossa the squamosal posterolaterally and the opisthotic medially, but its medial contact with the prootic is obscured. Together with the prootic it forms a large foramen stapedio-temporale. The quadrate forms a poorly developed processus trochlearis oticum that is composed of a rugose area.

##### 

**Epipterygoid** The epipterygoids are not exposed in either specimen.

##### 

**Pterygoid** The pterygoids are almost intact in PMOL-SGP A0100-3 except for their anteriormost edges (Figures 4B and 5A-B). The pterygoid has a long posterior process reaching as far as the back of the skull and terminating slightly anterior to the basioccipital-basisphenoid suture. The pterygoid covers the cranioquadrate space and contacts the posterolateral corner of the basisphenoid but not the basioccipital. The pterygoid has a short dorsal contact with the exoccipital, but this contact is not part of the skull surface. The foramen posterius canalis carotici interni opens at the back of the skull within the ventral surface of the pterygoid. The quadrate ramus of the pterygoid bears a well-developed, oval-shaped pterygoid fossa. The processus pterygoideus externus is present and it is characterized by a posteriorly extending horizontal plate and a dorsoventrally thickened vertical plate. A characteristic feature of the pterygoid is a large oval opening just anterior to the basisphenoid and posterior to the region where the pterygoids meet one another along the midline. This opening has intact margins, is clearly not a result of erosion or any other taphonomic processes, but is distinct from the foramen posterius canalis carotici palatinum. We interpret this structure as the remnant of the interpterygoid vacuity. Anterolaterally, the pterygoid bears a margin that is indicative of a large foramen palatinum posterius.

##### 

**Supraoccipital** Much of the crista supraoccipitalis is displaced and preserved in fragments in PMOL-SGP A0100-1 (Figures 4 and 5A). The supraoccipital provides only a small contribution to the skull roof where it contacts the parietals. The ventral plate of the supraoccipital contacts the opisthotic laterally and forms the dorsal margin of the foramen magnum. The crista supraoccipitalis extended apparently only slightly beyond the posterior tip of the squamosals. In PMOL-SGP A0100-3 the supraoccipital crest is intact as exposed in ventral view and does not protrude much beyond the level of the occipital condyle.

##### 

**Exoccipitals** The exoccipitals are preserved on both sides in PMOL-SGP A0100-3 (Figures 4 and 5A). They form the ventrolateral wall of the foramen magnum. A pair of foramen nervi hypoglossi pierce each elements but the formed foramen jugulare posterius is not distinct from the fenestra postotica (Figure 5A). Laterally, the exoccipitals contact the opisthotic and have a ventromedial contact with the basioccipital. Anteroventrally, the exoccipital has a short contact with the posteriormost tip of the pterygoid, but this contact does not contribute to the smooth, palatal surface of the skull. A suboval, unossified area excludes the exoccipital from anteromedially contacting the basisphenoid.

##### 

**Basioccipital** The basioccipital has a pair of basioccipital tubera with rounded posterior edges that extends as a roof over the foramen nervi hypoglossi when the skull is viewed ventrally (Figures 4B and 5A-B). The neck of the basioccipital condyle is short and lacks paired ridges or grooves. The basioccipital has no contact with the pterygoid and the processus interfenestralis of the opisthotic is therefore visible in ventral view. Anteriorly, the basioccipital meets the basisphenoid via a transverse suture. A shallow concavity extends on the ventral surface of the basioccipital that barely protrudes onto the basisphenoid.

##### 

**Prootic** The prootic is barely exposed on the right side of the skull of PMOL-SGP A0100-1 (not visible in dorsal view due to crushing). The skull roof in this specimen is deformed and thereby obscures the dorsomedial third of the ear capsule. On the left side, the ear capsule is so crushed that the structures cannot be identified with confidence. The prootic contributes to the large foramen stapedio-temporale together with the quadrate and maybe even with the opisthotic. There seem to be no prootic contribution to the processus trochlearis oticum (i.e. the rugose surface on the anterodorsal portion of the quadrate) by the prootic.

##### 

**Opisthotic** The opisthotic is exposed in both skulls (Figures 4 and 5A-B). The supraoccipital has a thin lateral lamina that partially covers the opisthotic within the upper temporal fossa. The opisthotic has a long lateral contact with the quadrate, may have a short contact with the squamosal, and ventrolaterally contacts the exoccipital. The dorsal portion of the opisthotic has a sutured contact with the quadrate whereas its contact with the squamosal is covered by matrix. The opisthotic forms a pillar-like processus interfenestralis that is visible in ventral view.

##### 

**Basisphenoid** The basisphenoid is preserved in good condition in PMOL-SGP A0100-3 (Figures 4B and 5B). It contacts the basioccipital along a straight suture posteriorly and is surrounded by the pterygoid rami laterally. The ventral surface is smooth and paired pits are therefore absent. The basisphenoid has a marked basipterygoid process in a form of a triangular, flat, horizontal plate that is sutured to and fits into a slightly-raised “pocket” of the pterygoid (Figure 4B). The foramen posterius canalis carotici interni is limited to the pterygoid but at least the anterior half of the floored canalis carotici interni extends along the pterygoid-basisphenoid suture. More anteriorly the carotid artery was exposed in a relatively deep and short, anteromedially directed sulcus, the fenestra caroticus, in the basisphenoid in which the split of the cerebral and palatine arteries was located. The cerebral branch diverged anteromedially and reentered the skull via the foramen posterius canalis carotici cerebralis at the medialmost corner of the fenestra. After exiting the fenestra, the palatine branch extended anteriorly in a shallow groove and entered the skull via the foramen posterius canalis carotici palatinum, which is situated on the basisphenoid-pterygoid contact just lateral to the residual interpterygoid vacuity. Since the latter foramen is clearly present, we infer that the palatine branch entered the skull here and not via the interpterygoid vacuity. The reduced condition of the interpterygoid vacuity in PMOL-SGP A0100-3 could represent a transitional state between a fully formed interpterygoid vacuity as seen in basal turtles [19,35,86,87] and a completely closed one as seen in numerous crown-group turtles [3].

#### ***Mandible***

The elegant and shallow mandible is preserved in articulation in PMOL-SGP A0100-3 (Figure 4B and 5B-C) and PMOL-SGP A0100-1 (Figure 4A), the former exposing the left coronoid region and the lateral plate of the dentary whereas the latter exposing the entire ventral and lateral aspects.

The dentary is characterized by a narrow triturating surface and a fused symphysis but neither the dentary ridges nor the anterodorsal tip of the symphyseal region are exposed. Laterally, the dentary extends posteriorly to meet the angular and the surangular whereas its contact with the articular is uncertain.

The coronoid is rather low and a long. An anteriorly tapering splenial is present that extends below the Meckelian canal along the dentary and approaches the symphysis. The splenial sends a posterior process between the angular and the prearticular, whereas the angular sends a similarly long anterior process into the splenial ventral to this projection. At the anteroventral tip of the angular process there is a triple junction with the dentary and the splenial. The processus retroarticularis is short. The splenial has a short dorsal contact with the coronoid.

#### ***Hyoid apparatus***

Both cornu branchiale I are preserved almost in situ in PMOL-SGP A0100-3 (Figures 4B and 5A-B), the right one being slightly crushed and incomplete. There is no evidence of an ossified corpus hyoidis or cornu branchiale II and these structures were therefore likely cartilaginous. The cornu branchiale I is a single, elegant element that can be divided into an anterior horizontal half and a posterior vertical half. It tapers posteriorly and terminates in a narrow, whip-like structure. When the skull is viewed from laterally, the border of the vertical and the horizontal portion is roughly at the level of the posterior rim of the cavum tympani.

#### ***Shell***

##### 

**Carapace** The carapace is present in all three specimens (Figures 3, 6 and 7A). PMOL-SGP A0100-1 and PMOL-SGP A0100-2 cover the anterior third of the carapace of PMOL-SGP A0100-3. PMOL-SGP A0100-1 has a posteriorly incomplete and slightly anterodorsally compressed carapace whereas PMOL-SGP A0100-2 is considerably deformed along its long axis and its right lateral third is missing due to damage that occurred during recovery of the block (see Materials and Methods). PMOL-SGP A0100-3 is not deformed; the exposed portion preserves the original outline of the carapace suggesting a relatively wide shell.

##### 

**Carapacial bones** The nuchal is a trapezoidal element and more than twice as wide than long (Figure 6). The nuchal emargination is minor in PMOL-SGP A0100-1 but appears to be slightly deeper in PMOL-SGP A0100-2. This emargination extends onto peripheral 1 in both specimens.

There are eight pairs of costal bones, all of which have firm contacts with the peripherals and lack costal fontanelles. The reduction of neural 7 in PMOL-SGP A0100-2 allows for a short, medial contact of costals 7, which contrasts the morphology of PMOL-SGP A0100-3, where a subdivided neural 7 does not allow for this contact (Figures 6B and 7A). This region is not preserved in PMOL-SGP A0100-1.

Costal 1 tapers laterally and is subequal in anteroposterior length with the more posterior costals. The figure of PMOL-SGP A0100-2 (Figure 6B) hints at a seemingly longer costal 1, but this is an optical illusion resulting from distortion. Costal 2 has a slightly concave anterolateral outline and sends a wide rectangular posterolateral process into the posterior half of peripheral 4, as is best seen on the left side of PMOL-SGP A0100-1 (Figure 6A), but also visible on the right side and in PMOL-SGP A0100-2 (Figure 6B). Costal 3 is the mediolaterally widest element and has straight and parallel anterior and posterior sides. Costal 4 is slightly concave anteriorly and convex posteriorly and has strongly concave contacts with peripheral 6 and 7. Costal 5 is slightly convex anteriorly and posteriorly. It has a short, oblique contact with peripheral 7 and a strongly concave contact with peripheral 8. PMOL-SGP A0100-3 is different in that costal 5 barely touches peripheral 7 (Figure 7A). The contact of costal 6 with peripheral 8 projects more laterally relative to its contact with peripheral 9. Costal 7 has an oblique and straight anterior border and a concave posterior border. Costal 8 is narrow and slightly convex anteriorly and posteriorly.

The neural series is complete and consists of eight elements, including a subdivided neural 7 in PMOL-SGP A0100-3 (Figure 7A). In PMOL-SGP A0100-2 the series is interrupted by the short contact of costals 7 (Figure 6B). In PMOL-SGP A0100-1, this region is incomplete. Most neurals are hexagonal, coffin-shaped elements with the short sides facing anterolaterally. Neural 1 of PMOL-SGP A0100-1 and 3 is quadrangular whereas it is hexagonal with short sides facing posterolaterally in PMOL-SGP A0100-2 the quadrangular element being neural 2 instead. In PMOL-SGP A0100-3 neural 7 is subdivided into a larger, regular, hexagonal element and a small, square element and neural 8 is hexagonal. In PMOL-SGP A0100-2 both neurals 7 and 8 are pentagonal and do not contact one another, thereby allowing for a medial contact of costals 7.

There are two suprapygals, the anterior one is trapezoidal has no contacts with the peripherals and considerably wider and shorter in PMOL-SGP A0100-2 than in PMOL-SGP A0100-3 (Figures 6B and 7A). Suprapygal 2 is a wide element that contacts costal 8 and peripheral 11.

There are 11 pairs of peripherals (Figures 6 and 7). A distinct gutter extends from the lateral corner of the nuchal to peripheral 7 along the lateral margin of the carapace. Peripheral 1 contacts costal 1 and is larger on the right side than on the left in PMOL-SGP A0100-1 (Figure 6A). Peripheral 2-6 are narrow elements whereas 7-11 are considerably expanded laterally. Peripheral 8 is the widest peripheral element and has a strong medial projection into costal 5 in all specimens.

##### 

**Carapacial scales** There are five vertebrals and a single wide cervical (Figures 6 and 7B). Vertebral 1 is wider than long and barely touches peripheral 1. The proportions of vertebrals 2-4 vary somewhat among the specimens, but all are narrower than most pleurals. PMOL-SGP A0100-1 has the relatively widest vertebrals of all. Vertebral 2 is slightly longer than wide in PMOL-SGP A0100-3 (as reconstructed) and PMOL-SGP A0100-2 whereas in PMOL-SGP A0100-1 it is markedly wider than long. Vertebral 3 is slightly wider than long in PMOL-SGP A0100-3 and PMOL-SGP A0100-3-3 and slightly longer than wide in PMOL-SGP A0100-2. Vertebral 4 is considerably wider than long in PMOL-SGP A0100-3 and PMOL-SGP A0100-3-3 and this is less distinct in PMOL-SGP A0100-2. The vertebral 3-4 sulcus has anterior projection at the midline that extends onto the posterior portion of neural 5 in all specimens. Vertebral 5 is wider than long, contacts peripherals 11 laterally, and does not prolong onto the suprapygal (as preserved in PMOL-SGP A0100-1 and PMOL-SGP A0100-3).

The pleurals are all wider than long except for pleural 4 that is longer than wide (Figures 6 and 7A).

The marginals are either restricted to the peripherals or their borders coincide with the costo-peripheral contacts or, as in the case of marginals 5 and 7, they slightly lap onto the costals. Marginal 11 indistinctly prolongs onto the costals on the left sides of all specimens (right side not preserved in PMOL-SGP A0100-1).

##### 

**Plastron** The plastron is only exposed in PMOL-SGP A0100-3 (Figure 7B). In this specimen the plastron is preserved in perfect condition except for minor damage in the right bridge area. The dorsal aspect of the plastron is not visible. The plastron is characterized by complete ossification (i.e., no fontanelles) and compact, sutural contacts. Scale sulci are clearly developed. The anterior lobe is about 40% wider than long, shorter than the posterior lobe, and has a slightly rounded anterior margin. Mesoplastra are absent. The posterior lobe is posteriorly tapering, slightly wider at its base than long, and lacks an anal notch. At least one musk duct foramen is present between peripheral 4 and the hyoplastron.

##### 

**Plastral bones** The epiplastron is trapezoidal, shows a roughly transverse suture with the hyoplastron, an anteromedially directed contact with the entoplastron, and a sagittal contact with the other epiplastron (Figure 7B). The entoplastron is oval-shaped, about twice as long as wide, and only partially separates the epiplastra. The buttress of the hyoplastron is relatively low and it terminates on the anterior half of peripheral 2. The contact of the plastron with the carapace is tight but we interpret it as being ligamentous rather than sutured owing for the presence of plastral pegs. However, we note that our meaning of ligamentous contact is probably different from the concept of earlier studies [13,14]. The edges of the bridge peripherals slightly overlap the margin of the bridge of the plastron. Peripherals 3, 4, and the anterior third of peripheral 5 contact the plastron via well-developed pegs. Further posteriorly, the contact between the plastron and the carapace transfers into a smooth-edged contact until the posterior third of peripheral 6. Just medial to this edge the plastron is notched at the contact of the hyo-and the hypoplastron, but this space is filled up with two elements (on one side) that appear to be aberrant extra ossifications that meet the hyo- and the hypoplastron along finely serrated edges. The anterior edge of these elements is partially fused with the hyoplastron. More posteriorly, the hypoplastron contacts the peripherals via pegs with the inguinal buttress terminating on the anterior third of peripheral 8. The xiphiplastra are well developed and they have a fork-like contact with the posterolateral portion of the hypoplastron in ventral view. Interfingering interplastral sutures are absent.

##### 

**Plastral scales** One pair of gulars and one pair of extragulars are present. The gulars do not extend onto the entoplastron and the extragulars have a transverse contact with the humeral scales. The midline sulcus of the plastron is straight instead of sinusoidal. The pectoral scale is shorter than the abdominal. The femoral/anal sulcus is omega-shaped and the anals barely extend near the midline onto the hypoplastron. Four pairs of inframarginals are present, of which the third covers the hyo/hypoplastral suture (Figure 7B).

#### ***Appendicular skeleton***

The articulated distal half of the right fore limb of PMOL-SGP A0100-1 is exposed in antipalmar view next to the skull of PMOL-SGP A0100-3 (Figures 7B and 8B). The left distal fore limb of this individual is less complete and preserved tucked on the other side of the slab next to the carapace (Figure 6A). The articulated left fore and hind limbs of PMOL-SGP A0100-2 (Figure 6B) are preserved in palmar and antipalmar view, respectively (Figures 8C-D). Only the left ulna, radius, an associated phalanx, the distal end of the right humerus, and both femora are exposed in specimen PMOL-SGP A0100-3 (Figure 7B). An additional, isolated left hind limb is present on the slab that likely belongs to a fourth specimen (Figure 3).

##### 

**Humerus** The humerus has a slightly curved shaft with a suboval cross-section (Figure 8C). The lateral process is slightly better developed than the medial process and both processes are situated at the same level relative to one and another along the proximal part of the humerus. The ectepicondylar foramen is closed.

##### 

**Radius and ulna** The radius is elegant and narrow and has a straight and relatively flat shaft in cross-section (Figures 8B-C). Its proximal epiphysis is subcircular in cross-section whereas its distal epiphysis is expanded and more compressed. The articulation surface for the medial centrale extends along the distal margin of the epiphysis as in *Podocnemis expansa*[19]. The medial edge of the distal epiphysis lacks a medial projection that is otherwise present in *Macrochelys temminckii*. The lateral ridge below the proximal epiphysis, presumably for the attachment of the radio-ulnar ligament [19], is reduced. The ulna is flattened and more robust than the radius. The medial margin of the shaft is more curved than the lateral one. The ridge for the bicipital tendon attachment is reduced and the olecranon is poorly developed. The medial process of the proximal epiphysis is situated slightly below the level of the olecranon as in *M*. *temminckii* but unlike in *P*. *expansa*. The relative proportions of the proximal and distal epiphyses more resemble *M*. *temminckii* in having similar width (the distal being slightly wider).

##### 

**Manus** The relatively elongate and narrow phalanges of the manus suggest intermediate aquatic adaptation [88]. The phalangeal formula is 2-3-3-3-3 (Figures 8B-C). The unguals are clawed, narrow, and pointed, and decrease in size from the digit I to V. The distal articulation surfaces of the proximal phalanges exhibit posteriorly projecting flanges that underlap the proximal epiphysis of the preceding metacarpals. The first metacarpal is the shortest and the most robust. The lateral overlapping of the metacarpals with one another is present but not marked. The distal carpals are ovoid and that of the first digit is slightly wider than those of the remaining digits. There is a small pisiform and the medial centrale is tightly connected with the lateral centrale. The intermedium is not elongate proximodistally, the ulnare is flat and deep, and the radiale bears little if any articulation with the radius.

##### 

**Femur** The femur has a slightly curved shaft (Figures 7B and 8D). The trochanter minor faces anteriorly, the trochanter major faces dorsally, and the femoral head only slightly extends above the trochanters. The trochanters are moderately developed. The proximal epiphysis has a similar width as the distal one.

##### 

**Tibia and fibula** The tibia has a wide proximal epiphysis (Figure 8D) than *Podocnemis expansa* or *Macrochelys temminckii*. The ridge for the patellar tendon attachment is placed close to the midline of the shaft as in *M*. *temminckii* and unlike in *P*. *expansa* where it is shifted laterally. The fibula is straight and has a more expanded and more compressed distal epiphysis than its proximal one. Proximally, the shaft lacks a medial flange, unlike in *Podocnemis expansa*.

##### 

**Pes** The hooked fifth metatarsal is a large, blocky element (Figure 8D). The astragalus is fused with the calcaneum. The pedal formula is 2-3-3-3-? and digits 1-4 were clawed, whereas digit 5 is incompletely preserved. The first metatarsal is more robust than the others.

#### ***Vertebral column***

Four cervicals are preserved in PMOL-SGP A0100-2, three in PMOL-SGP A0100-1, and PMOL-SGP A0100-3 exhibits one cervical vertebra and two anterior caudals (Figures 4A, 6 and 8A).

In PMOL-SGP A0100-2 three cervicals are well exposed in lateral and dorsal views that could represent any series of cervicals between 2 to 6 (Figure 8A). In PMOL-SGP A0100-1 cervicals 2 and 3 are exposed in dorsal view (Figure 4A). The centra are amphicoelous and more than twice as long than high (excluding the ventral keel and including the dorsal spine). A low ventral keel extends along the entire midline of the centra. The transverse processes are compressed, relatively robust, with parallel anterior and posterior sides, and exhibit clear diapophyses. The transverse process does not extend much laterally and is slightly longer than wide. The posterior third of the transverse process extends beyond the middle of the centrum whereas its anterior two-thirds extend anteriorly to the middle of the centrum, terminating well before the anterior end of the centrum. The cervicals have well-developed bifurcated ribs. The dorsal articulation of the ribs with the transverse processes is not preserved and elements therefore must have shifted, but the anterior contact with the parapophysis is still preserved. The parapophyses are situated at the anteroventral margin of the centrum and is best developed in the second cervical preserved in PMOL-SGP A0100-2 (probably cervical 3 or 4). The neural arch is longer and more than twice as high as the centrum (centrum including the transverse process but excluding the ventral keel and the arch including the zygapophyses but excluding the dorsal spine). The postzygapophyses are only slightly separated and unite in a common low stem. The anterodorsal surface of the postzygapophysis is convex whereas the posterodorsal is concave with a groove extending anteromedially. The neural spines are damaged and their full height is therefore unknown, except for the most anterior preserved cervical in PMOL-SGP A0100-2. Cervical 2 has a long neural spine extending all along the dorsal surface of the arch whereas cervical 3 has a shorter spine (PMOL-SGP A0100-1). The anteriormost cervical in PMOL-SGP A0100-2 has a low but long spine, the following is higher, and the third has a short and high process. The prezygapophyses are a little higher than the postzygapophyses (except for the third preserved in PMOL-SGP A0100-2) and slightly extend beyond the level of the anterior edge of the centrum in lateral view.

## Results and discussion

### Taxonomic comments

**Figure 9:**
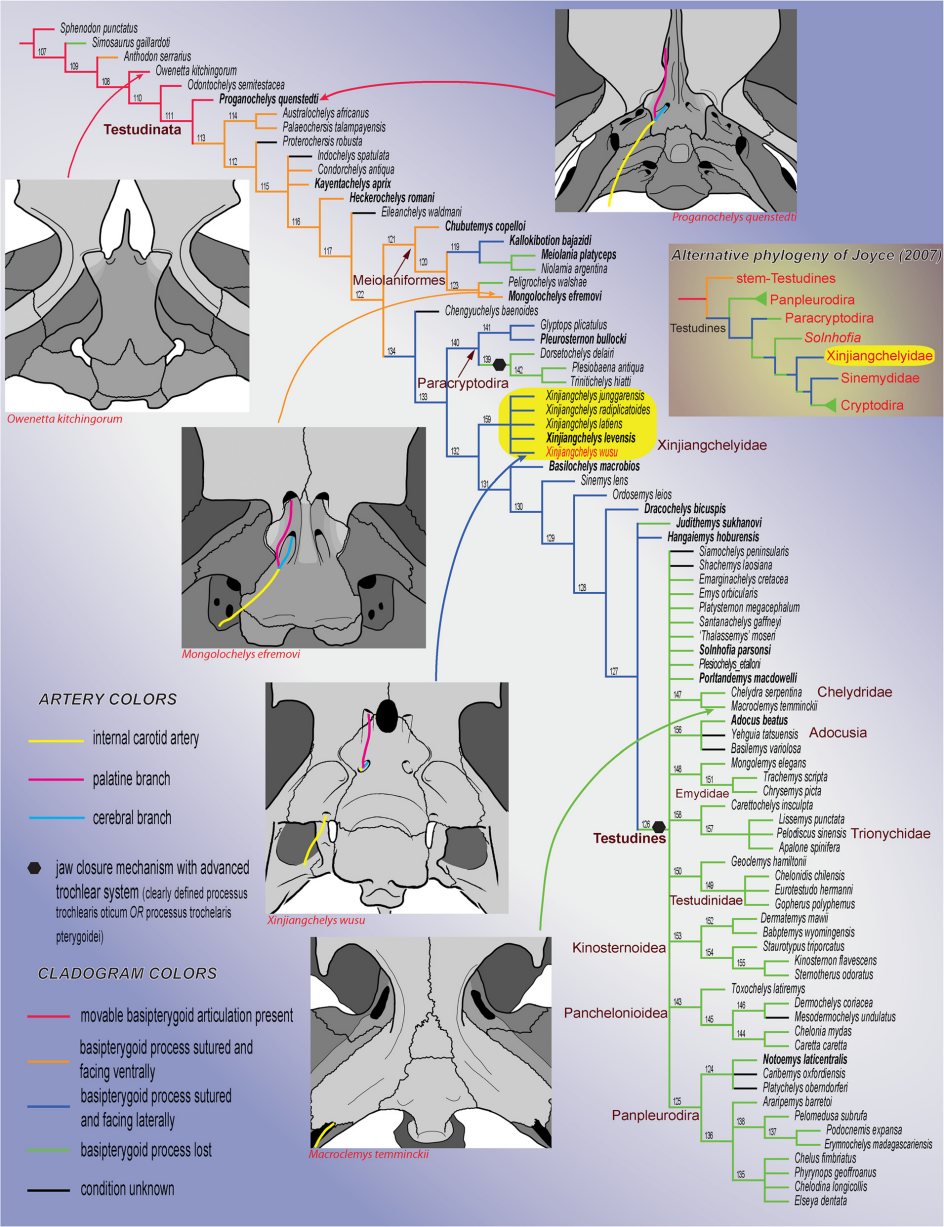
**Hypothetical relationships of the major clades of turtles and the evolution of the basipterygoid process and the carotid artery circulation system.** The cladogram is the strict consensus tree of 9261 trees of 870 steps obtained after a parsimony analysis of 237 morphological characters and 84 extinct and extant turtle taxa. The relationships of Durocryptodira [1] were constrained after the molecular phylogeny of Barley et al. [79]. Note the unorthodox position of Xinjiangchelyidae outside of Testudines. The more traditional phylogenetic placement of Xinjiangchelyidae [3] is presented on the right for comparison. Taxa in bold are figured in Figures 10, 11, 12. Numbers correspond to nodes.

Following the phylogenetic definition of Rabi et al. [59], *Xinjiangchelys wusu* is assigned to Xinjiangchelyidae because it is recovered in a monophyletic group together with *Xinjiangchelys junggarensis* (Figure 9). Other members of Xinjiangchelyidae include *X*. *radiplicatoides*, *X*. (*Annemys*) *latiens* and *X*. (*Annemys*) *levensis* and this clade is only supported by one unambiguous synapomorphy (Anal A:1, extension of anal scale onto hypoplastron).

Among taxa traditionally referred to Xinjiangchelyidae, the morphology of *X. wusu* is most similar to that of *X.* (*Annemys*) *levensis*, *Xinjiangchelys* (*Annemys*) *latiens* and *X. radiplicatoides*, however, a number of differences justify its recognition as a separate taxon. In contrast to *X*. *levensis*, the prefrontals do not meet in the midline in *X*. *wusu*, the basioccipital tubera are better developed, there are two foramina nervi hypoglossi instead of three, the vertebral 3-4 sulcus extends onto neural 5 not neural 6, and the midline plastral sulcus is straight instead of sinusoidal. *Xinjiangchelys* (*Annemys*) *latiens* has a proportionally more elongated skull, reduced frontal and jugal contribution to the orbit and sinusoidal midline plastral sulcus, whereas *X*. *radiplicatoides* has a more inflated skull, a slit-like interpterygoid vacuity instead of a round opening with very indistinct foramen caroticus palatinum, a strongly plicated carapace, and a sinusoidal midline plastral sulcus.

Since the interrelationships of xinjiangchelyids are unresolved in the consensus tree and pruning the rouge taxon *Xinjiangchelys junggarensis* reveals that *Annemys* (i.e., *X. levensis* and *X. latiens*) is paraphyletic (*levensis* forms the sister taxon of a *latiens*, *X*. *wusu* and *X*. *radiplicatoides* trichotomy), we suggest referring *wusu* and all other species to the genus *Xinjiangchelys* Ye 1986 [82] as this taxon has priority over *Annemys* Sukhanov and Narmandakh 2006 [58].

Recently, abundant remains of xinjiangchelyids were reported from the Mesa Chelonia turtle bone bed, which is stratigraphically situated 500 m below and spatially located 1 km away from the Turtle Cliff site [28]. These Mesa Chelonia turtles are represented by several partial skeletons and were all referred to an indeterminate species of *Annemys*[28]. The Mesa Chelonia form is very similar to *X. wusu* but a few differences are present and therefore we consider it a separate taxon. *Xinjiangchelys wusu* is about 15% larger, the foramen posterius canalis carotici interni is located along the posterior surface of the pterygoid, not in a notch at the back of the skull, the vertebral 3-4 sulcus extends onto neural 5 (extends onto neural 6 in eleven specimens out of twelve in the Mesa Chelonia form) and the plastral pegs are visible even when the plastron is articulated with the carapace, whereas the pegs are mostly covered by the peripheral ring in the fully ossified specimens of the Mesa Chelonia forms. A further difference might be that *X*. *wusu* lacks any types of fontanelles in the carapace or the plastron whereas they are present in more than half of the specimens from Mesa Chelonia that appear to be adult-sized individuals.

Another closely related form, mostly known by the skull, has been reported from the Junggar Basin [15] and was referred to *Annemys* sp. The foramen posterius canalis carotici interni of this skull is located in a notch between the basisphenoid and the pterygoid (unlike *X*. *wusu*) and the lateral plate of the jugal lacks a posterodorsal process extending ventral to the postorbital [15]. On the other hand, the skull from the Junggar Basin is very similar to the Mesa Chelonia form and we tentatively refer them to the same, yet unnamed taxon.

### The homology of the basipterygoid process in Mesozoic turtles

**Figure 10:**
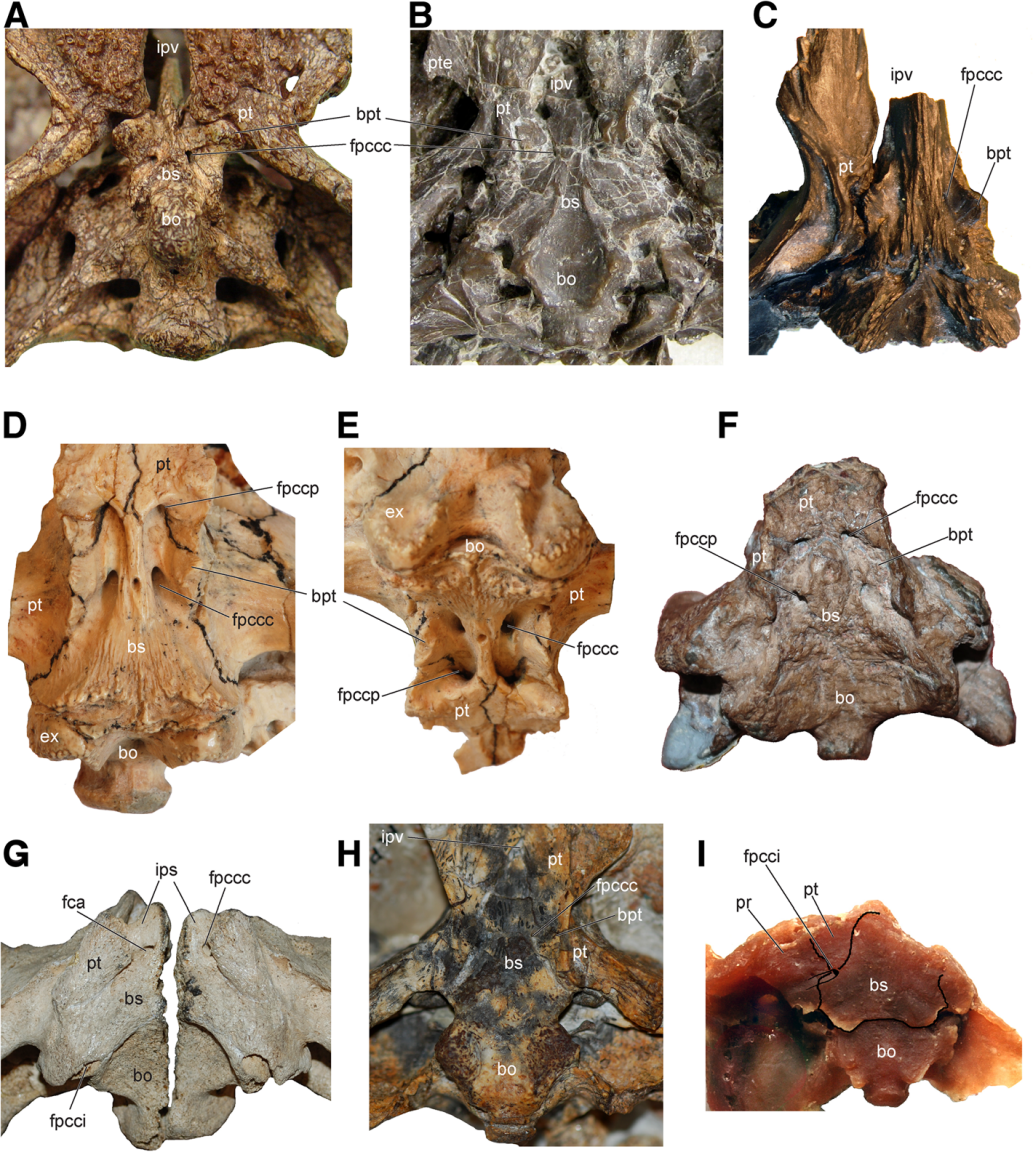
**Braincase and palatoquadrate of select basal turtles and a pan-pleurodire showing the presence or absence of a basipterygoid process. A**, *Proganochelys quenstedti* (SMNS 16980); **B**, *Kayentachelys aprix* (MCZ 8917); **C**, *Heckerochelys romani* (PIN 4561–2); **D-E**, *Mongolochelys efremovi* (PIN, uncatalogued) in ventral and oblique posterior view; **F**, *Kallokibotion bajazidi* (NHMUK R4925); **G**, *Meiolania platyceps* (NHMUK R682); **H**, *Chubutemys copelloi* (MPEF-PV1236); **I**, *Notoemys laticentralis* (cast of MOZP 2487). Abbreviations: **bo**: basioccipital, **bpt**: basipterygoid process, **bs**: basisphenoid, **ex**: exoccipital, **fca**: fenestra caroticus, **fpccc**: foramen posterius canalis carotici cerebralis, **fpcci**: foramen posterius canalis carotici interni, **fpccp**: foramen posterius canalis carotici palatinum, **ips**: intrapterygoid slit, **ipv**: interpterygoid vacuity, **pr**: prootic, **pt**: pterygoid, **pte**: processus pterygoideus externus.

Basal tetrapods and basal amniotes have no sutural relationship between their basicranium and the palatoquadrate region [89]. Instead, the basicranium articulates anteriorly with the pterygoid via the basipterygoid process of the basisphenoid (also termed the basitrabecular process) and posteriorly with the quadrate and the squamosal via the paroccipital process of the opisthotic. A basipterygoid process has been identified in a number of basal turtles and proto-turtles (Figures 10A-C), including *Odontochelys semitestacea*[48], *Proganochelys quenstedti*[19], *Palaeochersis talampayensis* Rougier et al., 1995 [90,91]*Australochelys africanus* Gaffney et al., 1994 [92,93], *Kayentachelys aprix*[86,94], *Heckerochelys romani*[35], and *Condorchelys antiqua* Sterli, 2008 [87,95]. Among this group of taxa, the more primitive ones, such as *O. semitestacea* and *Pr. quenstedti*, retain a movable basipterygoid articulation in the form of a ventrolaterally directed, blunt basipterygoid process that articulates with the corresponding facet in the pterygoid (Figure 10A). All more derived basal turtles with an unambiguous basipterygoid process are interpreted as having a fused articulation [18,20,35,66,86,87,93,94] whereas all more advanced stem-testudine taxa and all crown turtles are universally considered to have lost their basipterygoid process completely (e.g., [66]). Some derived taxa have nevertheless been hypothesized to retain a reduced basipterygoid process, but the homology of this structure has been a controversial issue.

**Figure 11:**
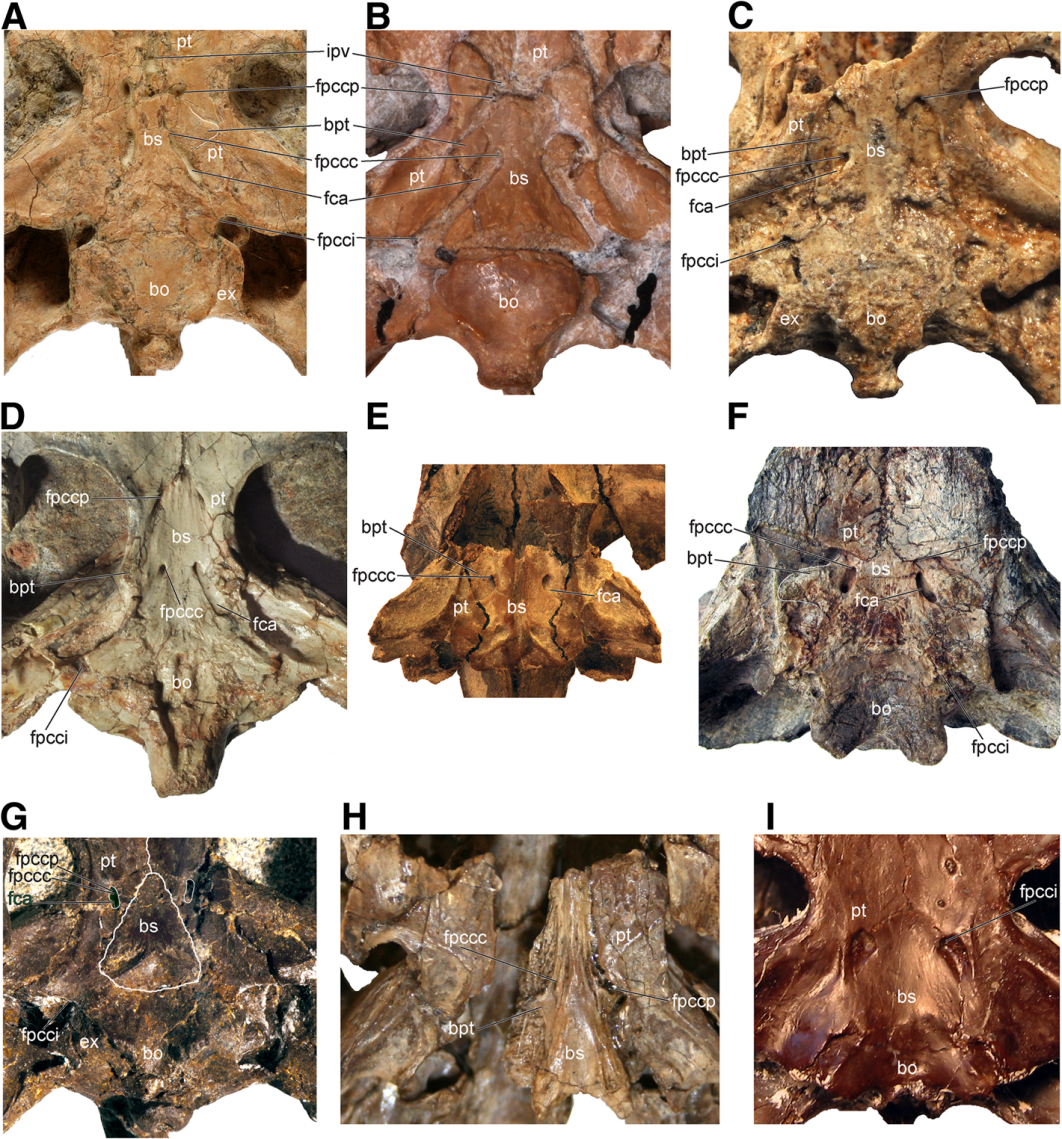
**Braincase and palatoquadrate of select Asian and North American Mesozoic turtles. A**, “*Annemys*” sp. from Turpan Basin, Mesa Chelonia, (SGP 2009/18, see [28]); **B**, *Xinjiangchelys levensis* (PIN 4636-4-2); **C**, *Sinemys gamera* (IVPP V9532-11); **D**, *Dracochelys bicuspis* (IVPP V4075); **E**, *Hangaiemys hoburensis* (PIN 3334-36); **F**, *Basilochelys macrobios* (MD8-2); **G**, *Judithemys sukhanovi* (TMP 87.2.1); **H**, *Pleurosternon bullockii* (UMZC 1041); **I**, *Eubaena cephalica* (MRF 571). Abbreviations: **bo**: basioccipital, **bpt**: basipterygoid process, **bs**: basisphenoid, **ex**: exoccipital, **fca**: fenestra caroticus, **fpccc**: foramen posterius canalis carotici cerebralis, **fpcci**: foramen posterius canalis carotici interni, **fpccp**: foramen posterius canalis carotici palatinum, **ipv**: interpterygoid vacuity, **pt**: pterygoid. *Judithemys sukhanovi* (G) has a reduced fenestra caroticus (fca, highlighted in green). The fpccp and the fpccc in this species are situated close to one another inside the fenestra caroticus and are therefore not visible in ventral view.

The presence of a basipterygoid process was first reported in the Late Jurassic turtle *Mesochelys durlstonensis* Evans and Kemp, 1975 [17], a taxon that was subsequently synonymized with *Pleurosternon bullockii*[96]. A similar structure was noticed by Gaffney (1979) [18] in *Glyptops plicatulus* Cope 1877 [97] and he concluded that it is not homologous with the unambiguous basipterygoid process of basal turtles based on topological considerations, a concept subsequently confirmed by Sterli et al. [20]. More recently, Brinkman et al. [15] identified a paired process of the basisphenoid similar to that seen in *Pleurosternon bullockii* (Figure 11H) in a broad selection of Jurassic and Early Cretaceous Asian eucryptodires and interpreted it as being homologous with the basipterygoid process of the earliest turtles, thereby contradicting the homology assessment of Gaffney [18] and Sterli et al. [20].

According to the homology concept of Gaffney [18] and Sterli et al. [20], the paired lateral processes of the basisphenoid that fit into corresponding pockets in the pterygoids in *G. plicatulus* and *Pl. bullockii* cannot be interpreted as the basipterygoid process because: a) they are placed posterior to the dorsum sellae and therefore have different topological relationships compared to the true basipterygoid processes seen in captorhinomorphs (e.g., the purported basal amniote condition) and b) because the processes in question do not ascend, as in basal turtles, but are instead aligned in the same horizontal plane as the pterygoids. Indeed, the basipterygoid process of captorhinomorphs is situated anterior to the dorsum sellae, the foramen posterius canalis carotici cerebralis [15], and the foramen nervi abducentis, whereas in *G. plicatulus* and *Pl. bullockii* the process in question is found posteriorly to these structures ([18], figure 23, note that the foramen posterius canalis carotici cerebralis is labeled foramen posterius canalis carotici interni). However, as already noted by others [15], when the condition seen in *Pr. quenstedti* (Figure 10A; unknown for Gaffney [18]) is compared to that of captorhinomorphs, it is evident that the dorsum sellae is in a derived position similar to that seen in *G. plicatulus* and *Pl. bullockii* (Figure 11H) in that it extends more anteriorly over the foramen anterius canalis carotici cerebralis ([19], figures 42-44). This anterior movement of the dorsum sellae likely resulted in the anterior migration of the foramen nervi abducentis and the foramen posterius caroticus cerebralis (the latter being erroneously named the foramen posterius canalis carotici interni in previous studies [17,18] for *G. plicatulus*, *Pl. bullockii*, and *Captorhinus* sp., as recently demonstrated [20,67]). The apparent morphocline shows that the basipterygoid process of *Pr. quenstedti,* whose homology relative to captorhinomorphs had never been questioned (e.g., [19]), is derived relative to the basal amniote condition and that it is in the same relative position as that seen in basal paracryptodires, except that in *G. plicatulus* and *Pl. bullockii* the cerebral foramen is positioned slightly more to the anterior. In addition, there is no reason to consider the foramina of the carotid circulation system to be stable landmarks that cannot shift from their position during evolution: in *K. aprix* the cerebral foramen is positioned just posteriorly to the basipterygoid process (Figure 10B) whereas in *H. romani* it is placed close to the anterior termination of the process (Figure 10C).

Sterli et al. [20] furthermore argued that the basisphenoid process of *G. plicatulus* and *Pl. bullockii* is not homologous with the basipterygoid process of basal amniotes, because it is directed laterally and found in the same plane as the pterygoid, unlike in *Pr. quenstedti*, where the basipterygoid process is directed ventrolaterally and situated ventral to the pterygoid. However, not all basal turtles have their basipterygoid process projecting ventrally. In *H. romani* the basipterygoid process is clearly present [35] but it projects laterally with a very minor ventral component and it is in the same plane as the pterygoid (Figure 10C). Thus, this taxon demonstrates that there was a phase in the evolution of the basicranium when the basipterygoid articulation was already sutured and was in the same level as the rest of the palate. The morphology of the basipterygoid in *H. romani* is close to that of xinjiangchelyids and “sinemydids/macrobaenids” (Figures 11A-E). A flat, triangular process projects laterally and slightly ventrally in these taxa to fit into the corresponding pit of the pterygoid in the same plane. There is no basis for interpreting this process as a neomorphic structure and given the identical topological position and the highly comparable shape the lateral basisphenoid process in basal paracryptodires (Figure 11H), xinjiangchelyids and “sinemydids/macrobaenids” can be confidently interpreted as being homologous with the basipterygoid process of basal turtles and basal amniotes.

### The basipterygoid process in Mesozoic turtles

**Figure 12:**
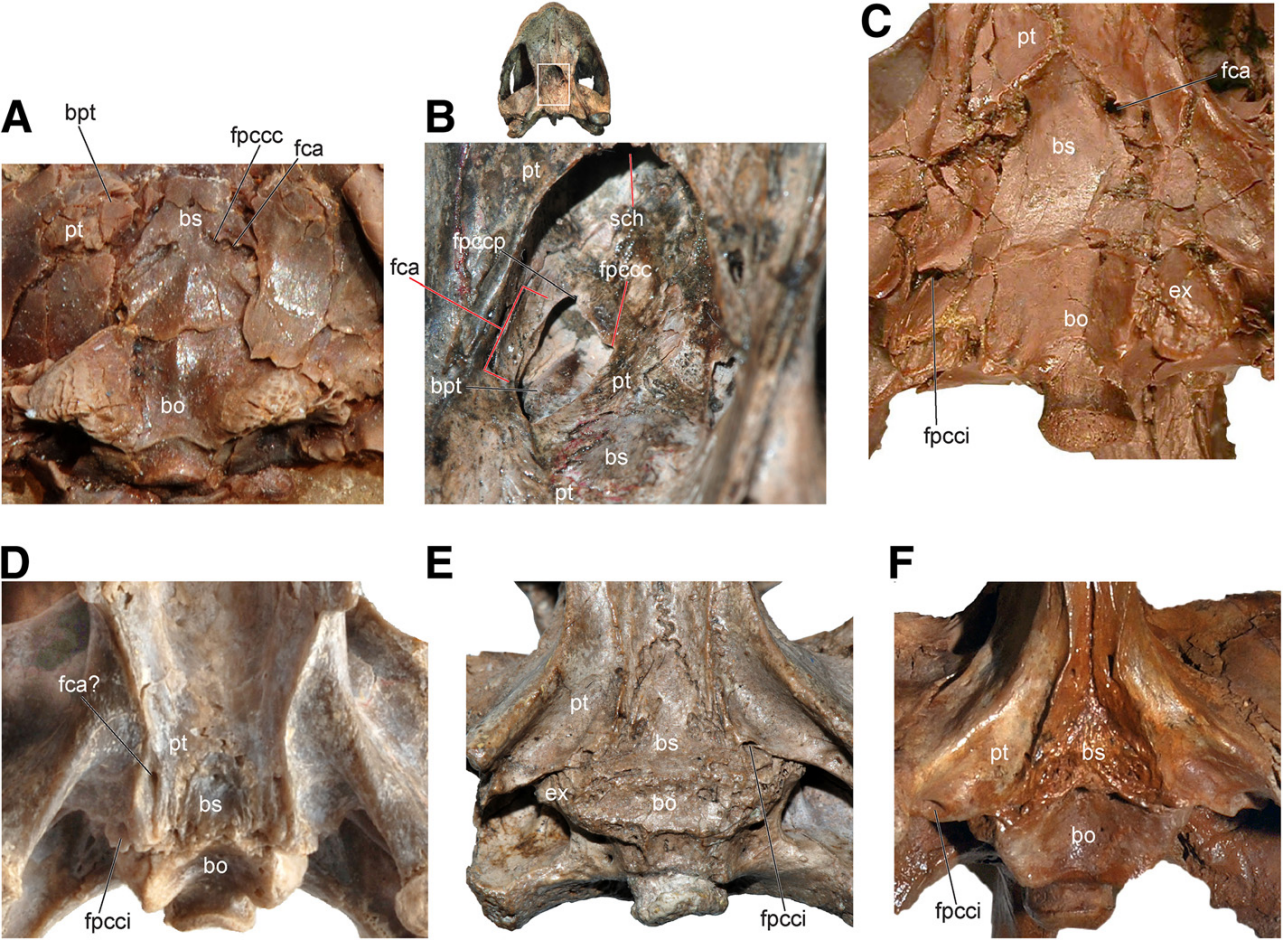
**Braincase and palatoquadrate of select Mesozoic turtles. A**, *Hoyasemys jimenezi* MCCM-LH-84; **B**, *Sandownia harrisi* (MIWG 3480); **C**, *Adocus* sp. (CCM 60-15); **D**, *Solnhofia parsonsi* (TM 4023); **E**, *Portlandemys mcdowelli* (NHMUK R2914); **F**, *Bouliachelys suteri* (SAM P41106). Abbreviations: **bo**: basioccipital, **bpt**: basipterygoid process, **bs**: basisphenoid, **ex**: exoccipital, **fca**: fenestra caroticus, **fpccc**: foramen posterius canalis carotici cerebralis, **fpcci**: foramen posterius canalis carotici interni, **fpccp**: foramen posterius canalis carotici palatinum, **pt**: pterygoid, **sch**: secondary choana. *Sandownia harrisi* (B) appears to retain a small basipterygoid process inside the fenestra caroticus, but given that is not exposed on the palatal surface of the skull and not visible in ventral view we suggest reflecting this difference in the scoring of this taxon in the future. *Adocus lineolatus* (C) has a reduced fenestra caroticus (fca). The fpccp and the fpccc in this species are situated close to one another deep inside the fenestra caroticus and therefore they are not visible in ventral view.

Since the basipterygoid process is generally interpreted to be a primitive character absent in derived turtles, many published descriptions of Mesozoic turtle skulls fail to report and illustrate the basipterygoid process. This is especially true for various Jurassic and Early Cretaceous Asian forms (i.e., xinjiangchelyids, sinemydids, and macrobaenids, Figure 11A-E). In addition to the taxa listed in a previous study [15] we further identified a laterally facing basipterygoid process in *Kallokibotion bajazidi* (Figure 10F), *Dracochelys bicuspis* (Figure 11F), *Manchurochelys manchoukuoensis*, *Sinemys brevispinus* (as also reported elsewhere [55]), *Ordosemys leios*, *Xinjiangchelys levensis* (Figure 11B), and *Xinjiangchelys latiens*, the alleged stem-adocusian *Basilochelys macrobios* (Figure 11F) and the basal eucryptodire *Hoyasemys jimenezi* (Figure 12A). In *Sandownia harrisi* the basipterygoid process is reduced and only visible in the floor of an opening formed by the pterygoids (i.e., the fenestra caroticus, Figure 12B). A similar morphology may be present in the macrobaenids *Judithemys sukhanovi* (Figure 11C) and *Macrobaena mongolica* and in the adocid *Adocus lineolatus* (Figure 12C) but the corresponding opening is so tight that the basipterygoid process (if any) is not visible. Consequently, we suggest scoring these taxa, including *S*. *harrisi*, as lacking the basipterygoid process, since the ventral surface of the basicranium lacks this structure. Various early marine turtles, including *Solnhofia parsonsi* (Figure 12D), *Portlandemys mcdowelli* (Figure 12E), *Plesiochelys etalloni*, and the early protostegid *Bouliachelys suteri* (Figure 12F) also lack basipterygoid processes. All other members of Testudines, including *Mongolemys elegans* lack a basipterygoid process as well.

The basipterygoid process is present and ventrolaterally directed in several representatives of the Meiolaniformes, a recently recognized Mesozoic to Pleistocene clade of basal turtles [16], including *Mongolochelys efremovi* (Figures 10D-E) and *Chubutemys copelloi* (Figure 10H). Another putative member of this clade, *Kallokibotion bajazidi* (Figure 10F) also retains the downward facing basipterygoid process (contrary to a previous report [98]). On the other hand, in *Meiolania platyceps* it is not the basisphenoid that extends ventrally to contact the pterygoid but rather it is the pterygoid that sends a process dorsally to contact the basisphenoid and to form the lateral wall of the intrapterygoid-slit ([99], figure 58). This is apparent since the suture between the basisphenoid and the pterygoid extends inside the fenestra caroticus, indicating that the basipterygoid process is lost (Figure 10G). A similar morphology can be observed in the Eocene meiolaniid *Niolamia argentina* as well. In the solemydid *Helochelydra nopcsai* the basipterygoid process is clearly absent given the complete loss of basisphenoid exposure whereas the condition in *Naomichelys speciosa* is clearly more derived than in more basal turtles (e.g. *Kayentachelys aprix*, Figure 10B) but a clear interpretation is difficult at the moment.

The oldest known panpleurodire skull is that of *Notoemys laticentralis* (Figure 10I) from the Late Jurassic of Argentina. The basisphenoid of this species shows a very reduced lateral protrusion just anterior to the foramen posterius canalis carotici interni ([100], Figure 2B; [101], pl. 1C). Since the split of the cerebral and palatine branches of the carotid artery is always situating ventral to the basipterygoid process in turtles known to retain this structure, we do not consider the protrusion of *Notoemys laticentralis* to be homologous with the basipterygoid process, given that it is situated dorsal to the split of the arterial branches, not ventral. The same rationale is applied for the interpretation of a lateral protrusion in the basisphenoid of several chelids and in *Araripemys barretoi* Price, 1973 [102-104].

Given that this structure has been notoriously overlooked in many Mesozoic taxa, we suggest that future workers should always explicitly note the presence or absence of the basipterygoid process while describing and/or scoring extinct turtles and also illustrate the basisphenoid accordingly. We suggest using the term “basipterygoid process” or “processus basipterygoideus” instead of “basitrabecular process” since the latter is less widely used in the fossil turtle literature. The term “fused basipterygoid articulation” [66] is not very precise since the basipterygoid process and the pterygoid are never fused per se, but rather connected by a suture.

### The evolution of the basipterygoid process in turtles

In the basal most known Triassic turtles and proto-turtles, such as *Proganochelys quenstedti* and *Odontochelys semitestacea*, the basipterygoid process is a robust and relatively thick structure that is directed ventrolaterally to articulate with a facet in the pterygoid. The pterygoid of these turtles is situated ventrally to the plane of the basisphenoid (Figure 10A). In spite of the presence of a kinetic joint in these taxa, their skull was not kinetic in the sense of others Holliday and Witmer [105]. In more derived turtles, such as *Palaeochersis talampayensis* and *Australochelys africanus*, the basipterygoid process is still prominent and faces ventrolaterally, but the articulation with the ventrally positioned pterygoid is transformed into a sutural contact. The Early and Middle Jurassic turtles *Kayentachelys aprix* (Figure 10B) and *Condorchelys antiqua* together with Cretaceous *Mongolochelys efremovi*, *Kallokibotion bajazidi* and *Chubutemys copelloi* (Figures 10D-F,G) represent a more advanced phase in that the process is more reduced and compressed, but the basisphenoid is still situated dorsal to the pterygoid. The next phase is exemplified by *Heckerochelys romani* (Figure 10C), and various members of Xinjiangchelyidae, Sinemydidae, and Macrobaenidae (Figures 11A-E) where the process is compressed and mainly laterally oriented and the basisphenoid is aligned with the pterygoid.

According to our phylogenetic hypothesis, the complete reduction and reorientation of the basipterygoid process happened independently in a number of turtle clades. The basipterygoid process was lost once within paracryptodires since basal members, such as *Glyptops plicatulus* and *Pleurosternon bullockii* (Figure 11H), still retain a process, whereas it is absent in *Compsemys victa* and all baenids (Figure 11I) [106-109]. The basipterygoid process is furthermore lost in derived members of Meiolaniformes (i.e., *Niolamia argentina*, *Peligrochelys walshae* and *Meiolania platyceps*, Figure 10G). At least one more independent loss occurred within crown Testudines (i.e., along the stem of Pleurodira and Cryptodira) as indicated by the presence of the basipterygoid process in most xinjiangchelyids, sinemydids, and *Hangaiemys hoburensis*. Furthermore, the basal position of *Judithemys sukhanovi* implies an additional independent loss in this species.

Considering the more traditional phylogenetic hypotheses that place Xinjiangchelyids on the stem of Cryptodira (e.g. [3]), these either infer two additional independent losses of the basipterygoid process (in Panpleurodires and early marine turtles including *Solnhofia parsonsi*) or alternatively (and perhaps less likely) the basipterygoid process was reacquired in basal paracryptodires (pleurosternids), xinjiangchelyids and sinemydids (Figure 9). Since our results themselves demonstrate that the loss of the basipterygoid process is quite homoplastic in turtles, two additional losses do not render considerably lower support for the traditional phylogenetic hypothesis [3] relative to the hypothesis presented here.

The reduction of the basipterygoid process in paracryptodires and crown-group Testudines was associated with the expansion of the parasphenoid ventral to the basisphenoid that eventually resulted in the complete enclosure of the arteries of the carotid circulation system in bone [20]. In the case of Cryptodires the pterygoid was involved as well [15,66]. In all groups the synchronous loss of the basipterygoid process led to the final reinforcement of the basicranial region [19,87].

The multiple parallel losses of the basipterygoid process suggest that several clades of turtles gained an advantage by reinforcing the contact between the basicranium and the palatoquadrate. Interestingly, the loss of the basipterygoid process is often associated with another derived trait, the presence of a well-developed trochlear system. Many pancryptodires, including all crown-group members, and all pleurodires have an advanced jaw closure mechanism where the jaw adductor muscle is redirected by the otic trochlea in the former and the pterygoid trochlea in the latter, in both cases acting like a pulley system [65]. As already pointed out previously [94,110], many basal taxa do not possess, or do not clearly possess the advanced otic trochlear process found in most crown-group cryptodires. Our review of taxa that retain a basipterygoid process, including basal turtles, most meiolaniforms, xinjiangchelyids, sinemydids, and macrobaenids reveals that these taxa possess poorly developed otic trochlea (if any) in form of a rugose surface or a low ridge that only barely protrudes anteriorly, unlike in taxa where the basipterygoid process is absent, including plesiochelyids, eurysternids, baenids, and most crown-group cryptodires, where the otic trochlea is robust and protrudes significantly (Figure 9). The condition in pleurodires is also consistent with this correlation as they have an advanced trochlear process formed by the pterygoid and the basipterygoid process is absent even in the earliest known extinct species, *Notoemys laticentralis* (Figures 9 and 10I) [101].

The loss of the basipterygoid process and the enclosure of the carotid circulation system in bone probably results in a reinforced connection between the basicranium and the palatoquadrate and therefore in a more rigid skull. As previous works pointed out [66,87,111] the development of advanced jaw closure mechanisms during turtle evolution likely required a more rigid skull that is compliant with higher bite performance and the loss of the basipterygoid process in association with the formation of an advanced trochlear system is therefore consistent with this pattern. In this regard the evolution of turtles parallels other amniote groups with rigid skulls including, therapsids, sauropterygians, and crocodyliformes which also lost their basipterygoid processes and enclosed the carotid system during the reinforcement of the basicranium [89].

### Phylogenetic implications

The phylogenetic analysis found 9261 most parsimonious trees (length = 870) and most cryptodire clades were only partially recovered in the strict consensus relative to the molecular based topology we used as a constraint. This might be due to character conflict caused by the extinct taxa designated as floaters (see Appendix B for a list of taxa) and urges a thorough review of all scorings of the matrix in the future.

The results of our analysis conflict with previous studies regarding the position of Xinjiangchelyidae (i.e., the clade of all turtles more closely related to *Xinjiangchelys junggarensis* than to any extant turtle), a group that is otherwise commonly hypothesized to be pancryptodiran [1-4,8-11,16,61,70,72], by placing it outside of crown group Testudines (Figure 9). On the other hand they are consistent with the results of the most recent analysis of turtle phylogeny [59]. Xinjiangchelyids indeed possess a number of primitive characters, including the presence of a reduced interpterygoid vacuity and a basipterygoid process, the absence of a bony canal for the split of the cerebral and palatine branches, the presence of dorsal processes of epiplastron, long first dorsal ribs, and amphicoelous cervicals. We identify two characters that are responsible for the basal position of Xinjiangchelyidae in our cladogram. In contrast to our current and earlier analysis [59], the presence of a basipterygoid process was previously scored as unknown whereas the first dorsal rib was scored as short for *Xinjiangchelys junggarensis* (formerly *X*. *latimarginalis*), the only xinjiangchelyid in the original matrix [16]. However, as we demonstrated above and in accordance with a recent study [15], a basipterygoid process is present in the basicranium of *X*. *radiplicatoides*, *X*. *wusu*, *X. levensis* and *X*. *latiens*. The first dorsal rib of *X*. *latimarginalis* was previously identified as short [72] (reaching about half way to the axillary buttress), but revision of the specimen in question (IVPP V9537-1) reveals that the rib was long. In fact, the rib is incompletely preserved, but the corresponding scar extends along the entire anterior edge of the second dorsal rib. A long first dorsal rib is furthermore present in *X. levensis* (unknown for *X*. *latiens* and *X*. *wusu*) and a long scar is described and figured for *X*. *radiplicatoides*[15]. We therefore scored *X*. *junggarensis*, *X*. *radiplicatoides* and *X*. *levensis* as having a long first dorsal rib.

It was previously unknown that the junction of the palatine and cerebral branches of the carotid artery was not floored in xinjiangchelyids (see also [59]), but this can not be responsible for their basal position since sinemydids had been scored with this primitive condition [16], but were placed on the stem of crown Cryptodira.

On the other hand, we realized that the original matrix [16] contains a good number of inconsistently scored characters and fixing these errors would likely alter the current results (see also [59]). A comprehensive revision and expansion of this matrix is therefore in progress as part of a larger scale project (including WGJ, MR and J. Sterli).

#### ***Relationships of Cretaceous “Eucryptodires”***

The Cretaceous “eucryptodires” of Asia and North America, including *Sinemys lens*, *Ordosemys leios*, *Dracochelys bicuspis*, *Hangaiemys hoburensis*, and *Judithemys sukhanovi* are often collectively referred to as “sinemydids/macrobaenids” in the literature ([12] and references therein) reflecting the likely paraphyletic nature of the group (see Rabi et al. [59] for a phylogenetic definition of these clades). All previous works, however, agree that these taxa are placed somewhere along the stem of crown Cryptodira or that parts nest within it. Our cladogram here preliminarily places all of these turtles outside of Testudines in a paraphyletic grade more derived than Xinjiangchelyidae, but more basal than crown Testudines (Figure 9).

Previous analyses also acknowledged the presence of long first dorsal rib in *O. leios* and *D. bicuspis* but ignored the presence of a basipterygoid process in these taxa (see above), a condition that pulled them to a more basal position, together with *Hangaiemys hoburensis*, another taxon with a basipterygoid process but with short first dorsal rib. Interestingly, *Judithemys sukhanovi* lacks a basipterygoid process, but is found just outside of crown Testudines in a polytomy with *H*. *hoburensis*, which possess this process.

Some previous published analyses have actually found that some of these taxa do form a clade [3,4,16] but due to changes we introduced in their scorings, we cannot recover such groups rendering further support to the paraphyletic nature of “sinemydids/macrobaenids”.

#### ***Relationships of early marine turtles***

Most previous analyses that included various Late Jurassic marine European taxa (e.g., *Plesiochelys solodurensis*, *Portlandemys mcdowelli*, “*Thalassemys*” *moseri* Bräm 1965 [112]) and the Early Cretaceous South American *Santanachelys gaffneyi* variously united them into clades and/or paraphyletic grades somewhere along the stem of crown Cryptodira in a more basal position than xinjiangchelyids [3,4,10,61,70,113,114]. A recent exception is the analysis we modified in our study [16] and which found a (*Ples*. *solodurensis* + *Port*. *mcdowelli*) clade sister to Testudines and a (*Sol*. *parsonsi* (*Sant*. *gaffneyi* + “*T*.” *moseri*)) clade in a polytomy with members of “sinemydids/macrobaenids” on the stem of Cryptodira. We found these taxa in an even more derived position as part of the crown group Testudines (Figure 9). One of the reasons for the more derived position of these taxa is likely the recognition of the basipterygoid process in xinjiangchelyids and “sinemydids/macrobaenids” that is clearly lost in all the European taxa (Figures 10D-E) and *Santanachelys gaffneyi*. Again, these results must be viewed with caution given the necessity of a comprehensive revision of the current matrix (see above).

#### ***Relationships of Basilochelys macrobios***

*Basilochelys macrobios* from the Latest Jurassic/Earliest Cretaceous of Thailand has been hypothesized to represent an early crown group Cryptodire closely related to or nesting within Trionychia [61,115]. The position of *Basilochelys macrobios* in crown-group Cryptodira is not supported by our analyses and this taxon is recovered instead in the next less inclusive node to Xinjiangchelyidae outside of crown Testudines (Figure 9). *Basilochelys macrobios* has a sculptured shell surface that is reminiscent of certain nanhsiungchelyids [61] but there has been no attempt to homologize it with the sculpturing of trionychians and therefore we scored the type of sculpturing (Carapace E) as unknown. However, the scoring of the presence of “trionychian-type” sculpture (Carapace E-2) does not alter the position of *B. macrobios*. A trionychian-like sculpturing is also present in *Yehguia tatsuensis* and *Siamochelys peninsularis* ([1], MR, WGJ pers. obs.) but no matter how we score this character it does not influence the position of these three taxa. Based on detailed photographs we are confident that *B. macrobios* possesses a sutured basipterygoid process (Figure 11F; contrary what has been previously reported [61]) and this is clearly the reason for its relatively basal position in our analysis. *Y*. *tatsuensis* is recovered as part of Testudines in the Adocusia clade which is consistent with earlier hypotheses [1,16]. On the other hand, the placement of *S*. *peninsularis* in the crown group of turtles contrasts previous results [4,16].

## Conclusions

The discovery of *Xinjiangchelys wusu* from the Late Jurassic of the Turpan Basin, Xinjiang, China adds to the known diversity of xinjiangchelyid turtles and provides the first step in the direction of understanding the biogeographical relationships of the Turpan Basin tetrapod faunas with roughly coeval faunas, especially those of the adjacent Junggar Basin (e.g. [12]).

Xinjiangchelyids have been mostly known on the basis of shells but these new findings together with recently described material from the Junggar Basin provides new insights into the anatomy of the rest of the skeleton.

The study of *X. wusu* made clear the necessity for reviewing the basicranial morphology of Mesozoic turtles, which revealed that the basipterygoid process has been overlooked in a broad range of extinct taxa. The repeated independent loss of the basipterygoid process together with the enclosure of the carotid circulation system in bone during turtle evolution argues for strong selective pressures to reinforce the basicranial region and to develop a more rigid skull. Testing the phylogenetic implications of these novel anatomical data in a global context resulted in the unorthodox basal placement of xinjiangchelyids, sinemydids, and macrobaenids. This topology needs further testing since it would infer unexpected reversals in Pan-Pleurodires, including the reacquisition of a “reduced” mesoplastron and the reorganization of the entry of the carotid artery into the skull among others and therefore a thorough revision of the matrix is of primary importance. Nevertheless, this analysis, together with an earlier study [16], raises the issue that certain widely recognized Pan-Cryptodiran synapomorphies [91], including the complete flooring of the cranioquadrate space by the pterygoid and the presence of at least a poorly developed otic trochlea, might be symplesiomorphies of Testudines.

## Appendix A

### List of omitted taxa from the matrix of Sterli and de la Fuente (In press)

*Ninjemys oweni*, *Warkalania carinaminor*, *Patagoniaemys gasparinae*, *Otwayemys cunicularius*, *Prochelidella cerrobarcinae*, *Myuchelys latisternum*, *Chelodina colliei*, *Yaminuechelys maior*, *Dinochelys whitei*, *Neurankylus eximius*, *Boremys pulchra*, *Baena arenosa*, *Chisternon undatum*, *Macrochelys schmidti*, *Protochelydra zangerli*, *Chelonoidis gringorum*, *Stylemys nebraskensis*, *Echmatemys wyomingensis*, *Xenochelys formosa*, *Hoplochelys crassa*, *Plastomenus aff. thomassii*, *Anosteira ornata*.

## Appendix B

### List of taxa designated as floaters after constraining the relationships of Durocryptodira in the phylogenetic analysis

*Siamochelys peninsularis*, *Basilochelys macrobios*, *Ordosemys leios*, *Dracochelys bicuspis*, *Judithemys sukhanovi*, *Hangaiemys hoburensis*, *Xinjiangchelys junggarensis*, *Xinjiangchelys radiplicatoides*, *Xinjiangchelys wusu, X*. (*Annemys*) *latiens*, *X*. (*Annemys*) *levensis*, *Shachemys laosiana*, *Adocus beatus*, *Yehguia tatsuensis*, *Basilemys variolosa*, *Baptemys wyomingensis*, *Mongolemys elegans*, all members of Trionychidae and Panpleurodira, *Carettochelys insculpta*, *Toxochelys latiremis*, *Mesodermochelys undulatus*, *Plesiochelys etalloni*, *Santanachelys gaffneyi*, *Solnhofia parsonsi* and *Portlandemys mcdowelli.*

## Abbreviations

AM: Australian Museum, Sydney, Australia; CCM: Carter County Museum, Montana USA; FMNH: Field Museum of Natural History, Chicago, USA; IVPP: Institute of Vertebrate Paleontology and Paleoanthropology, Beijing, China; IWCMS: Dinosaur Isle Museum, see MIWG; MACN: Museo Argentino de Ciencias Naturales “Bernardino Rivadavia”, Buenos Aires, Argentina; MCCM: Museo de las Ciencias de Castilla–La Mancha, Cuenca, Spain; MCZ: Museum of Comparative Zoology, Harvard University, Cambridge, USA; MD: Sirindhorn Museum, Phu Kum Khao, Sahatsakhan, Kalasin Province, Thailand; MH: Naturhistorisches Museum, Basel, Switzerland; MIWG: Museum of Isle of Wight Geology, Sandown, United Kingdom; MNA: Museum of Northern Arizona, Flagstaff, USA; MOZP: Museo “Prof. Dr. Juan A. Olsacher”, Zapala, Argentina; MPEF: Museo Paleontológico Egidio Feruglio, Trelew, Argentina; MRF: Marmarth Research Foundation, Marmarth, North Dakota, USA; NHMUK: Natural History Museum of London, United Kingdom; PIN: Paleontological Institute, Russian Academy of Sciences, Moscow, Russia; PMOL: Paleontological Museum of Liaoning, Shenyang Normal University, Shenyang, China; SAM: South Australian Museum, Adelaide, Australia; SGP: Sino-German Cooperation Project; SMNS: Staatliches Museum für Naturkunde, Stuttgart, Germany; UMZC: University Museum of Zoology, Cambridge, United Kingdom; TM: Teylers Museum, Haarlem, The Netherlands; TMP: Royal Tyrrell Museum of Palaeontology, Drumheller, Canada.

## Competing interests

The authors declare that they have no competing interests.

## Authors’ contributions

WGJ initiated study and raised funds. SG obtained permits for fieldwork and MR, OW, CFZ, and WGJ participated in fieldwork. MR, CFZ, and WGJ gathered data, illustrated specimens, and performed analyses. MR wrote primary draft of manuscript. All authors read and approved manuscript.

## Supplementary Material

Additional file 1 Contains the taxon-character matrix used in the phylogenetic analysis in nexus. Click here for file

Additional file 2 Corresponds to the character-taxon matrix exported into a tnt. format file that can be analyzed with the TNT phylogenetic software. Click here for file
